# Oxidative stress-mediated PANoptosis and ferroptosis: Exploration of multimodal cell death triggered by an AIE-active nano-photosensitizer via photodynamic therapy

**DOI:** 10.7150/thno.111635

**Published:** 2025-06-09

**Authors:** Yuqing Wang, Chuxing Chai, Wangxing Lin, Juanmei Cao, Zhuoxia Li, Yifan Jin, Yiting Xu, Jianyu Zhang, Yong Qu, Jinshan Zhan, Tianqi Zhao, Yufan Chen, Meng Gao, Changzheng Huang, Min Li

**Affiliations:** 1 Department of Dermatology, Union Hospital, Tongji Medical College, Huazhong University of Science and Technology, Wuhan 430022, China.; Department of Clinical Nutrition, Union Hospital, Tongji Medical College, Huazhong University of Science and Technology, Wuhan 430022, China.; 2 Department of Hepatobiliary Surgery, Union Hospital, Tongji Medical College, Huazhong University of Science and Technology, Wuhan 430022, China.; 3 National Engineering Research Center for Tissue Restoration and Reconstruction, Key Laboratory of Biomedical Engineering of Guangdong Province, Key Laboratory of Biomedical Materials and Engineering of the Ministry of Education, Innovation Center for Tissue Restoration and Reconstruction, School of Materials Science and Engineering, South China University of Technology, Guangzhou 510006, China.; 4 Department of Dermatology, First Affiliated Hospital, Shihezi University, Shihezi 832008, China.; 5 Central Laboratory, Union Hospital, Tongji Medical College, Huazhong University of Science and Technology, Wuhan 430022, China.; 6 Department of Chemistry, Hong Kong Branch of Chinese National Engineering Research Center for Tissue Restoration and Reconstruction, The Hong Kong University of Science and Technology, Hong Kong 999077, China.

**Keywords:** Photodynamic therapy, Aggregation-induced emission, Oxidative stress, PANoptosis, Ferroptosis

## Abstract

**Background:** Aggregation-induced emission (AIE)-based photodynamic therapy (PDT) represents a promising strategy for cancer treatment for its capacity to activate specific cell death pathways through pronounced oxidative stress. While the activation of specific death pathways has been correlated with PDT efficiency and overall effect, the systematic coordination of oxidative stress across different cell death modalities to amplify therapeutic effects remains unexplored. Current research lacks systematic investigation into how oxidative stress coordinates multiple cell death pathways to amplify therapeutic outcomes of PDT.

**Methods:** We developed an AIE-based nano-photosensitizer (T-T NPs) to induce multimodal cell death through PDT. The system was characterized for mitochondrial targeting capability and reactive oxygen species (ROS) generation. Mechanistic analyses were conducted to evaluate programmed cell death pathways and ferroptosis induction in tumor.

**Results:** T-T NPs exhibited superior mitochondrial targeting and highly efficient ROS generation. This dual effect successfully triggered PANoptosis and ferroptosis. The synergistic activation of these pathways significantly enhanced PDT-mediated antitumor efficacy.

**Conclusion:** Our findings reveal that AIE-driven PDT can orchestrate multimodal cell death in tumor through oxidative stress modulation. By concurrently activating PANoptosis and ferroptosis, this approach establishes a novel paradigm for overcoming limitations of conventional single-pathway targeted PDT.

## Introduction

Photodynamic therapy (PDT) has emerged as a promising cancer treatment modality due to its non-invasive nature and minimal side effects. As a localized treatment, PDT has been applied to a wide range of tumors, including superficial skin tumors, head and neck cancers, esophageal cancer, extrahepatic cholangiocarcinoma, lung cancer, and bladder cancer, demonstrating considerable clinical potential 1-6. PDT operates by activating photosensitizers (PSs) with light at specific wavelengths, leading to the production of reactive oxygen species (ROS) and inducing oxidative stress, which damages cellular components and ultimately causes cell death 7-8. To enhance PDT efficacy, various novel PSs have been developed. Recently, PSs with aggregation-induced emission (AIE) properties have attracted significant attention due to their enhanced photostability and increased ROS generation, leading to greater tumor suppression compared to traditional PSs with aggregation-caused quenching (ACQ) effects 9-13.

The core antitumor mechanism of PDT involves the generation of ROS, causing oxidative stress and subsequent cell death 14. Various cell death mechanisms, such as apoptosis and necrosis, are closely tied to oxidative stress induced by PDT 15-16. More recently, novel cell death pathways like pyroptosis and ferroptosis have been observed in cancer cells undergoing PDT-induced oxidative stress 17-18. These unique forms of cell death can elicit strong immunogenic responses, thereby enhancing PDT's antitumor efficacy 19. For instance, Wang et al. 20 designed a membrane-targeted AIE-based PS, TBD-3C, which effectively induced pyroptosis and stimulated cancer immunotherapy through PDT. Similarly, Fang et al. 21 developed a multifunctional PS (CNTPA-TPA) that triggered ferroptosis and immune activation, leading to enhanced antitumor effects. Given the influence of cell death modalities on PDT's anticancer efficacy, strategies have emerged that combining PDT with cell death inducers, such as erastin (ferroptosis inducer) 22, decitabine (pyroptosis inducer) 23, and copper ions (cuprotosis inducer) 24, within a single nano-delivery system to activate specific cell death mechanisms. Most studies have examined the independent effects of these death pathways in PDT; however, recent research suggests that these pathways may interact synergistically, potentially amplifying antitumor effects. Initial studies have begun exploring dual cell death modalities, such as apoptosis-ferroptosis 25-26 and pyroptosis-ferroptosis 27. Our preliminary findings also intimate that cell death pathways may form a complex multimodal network in PDT, collaboratively enhancing antitumor efficacy 28. Based on these observations, we hypothesize that PDT can activate a multimodal cell death network through oxidative stress, achieving amplified antitumor outcomes and guiding the design of new PSs.

**Scheme 1:**
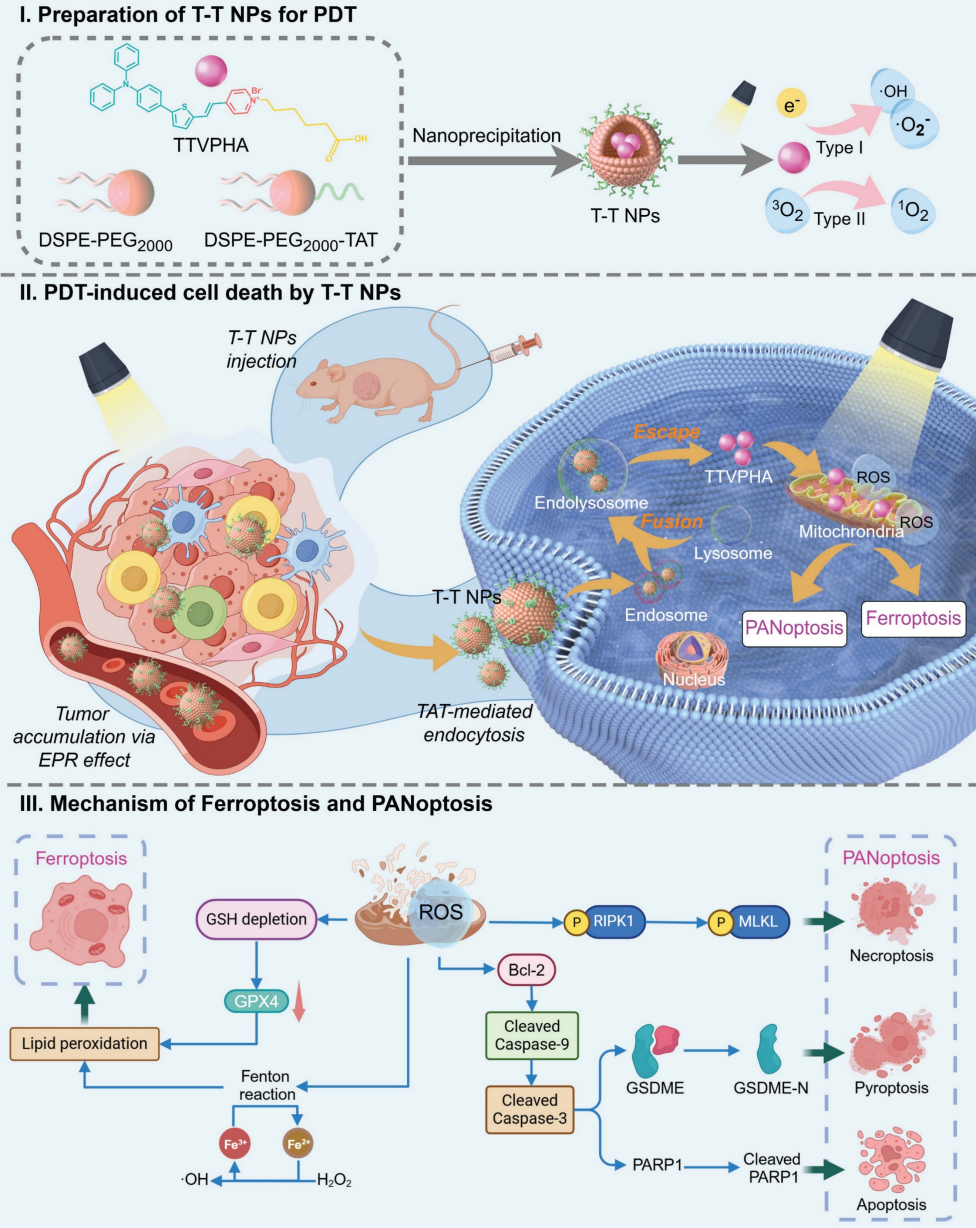
Schematic illustration of (I) preparation process and PDT pathway of T-T NPs; (II) tumor accumulation, cancer cell endocytosis, and cell death modalities of T-T NPs-mediated PDT; (III) mechanisms of ROS-induced multimodal cell death network through PDT of T-T NPs.

In this study, we developed a novel nano-formulated PS, T-T NPs, by encapsulating the mitochondria-targeted AIE-based PS TTVPHA within DSPE-PEG_2000_ and functionalizing it with TAT (a cell-penetrating peptide) to enhance cellular uptake. In both *in vitro* and *in vivo* models, we demonstrated that T-T NPs-mediated PDT produces substantial ROS in tumor cells, activating key factors involved in multiple cell death pathways, including PANoptosis (pyroptosis, apoptosis, and necroptosis) and ferroptosis across various cancers (melanoma and hepatocellular carcinoma), as illustrated in **Scheme 1**. To our knowledge, this study is the first to reveal that AIE PS-mediated PDT can trigger a complex network of multimodal cell death involving PANoptosis and ferroptosis, providing mutual reinforcement for enhanced antitumor effects. This multimodal strategy may offer a more comprehensive approach to cancer treatment with PDT, potentially overcoming resistance mechanisms, enhancing therapeutic efficacy, and simplifying the nanoformulation design for PSs.

## Results and Discussions

### Preparation and characterization of T-T NPs

**Figure 1:**
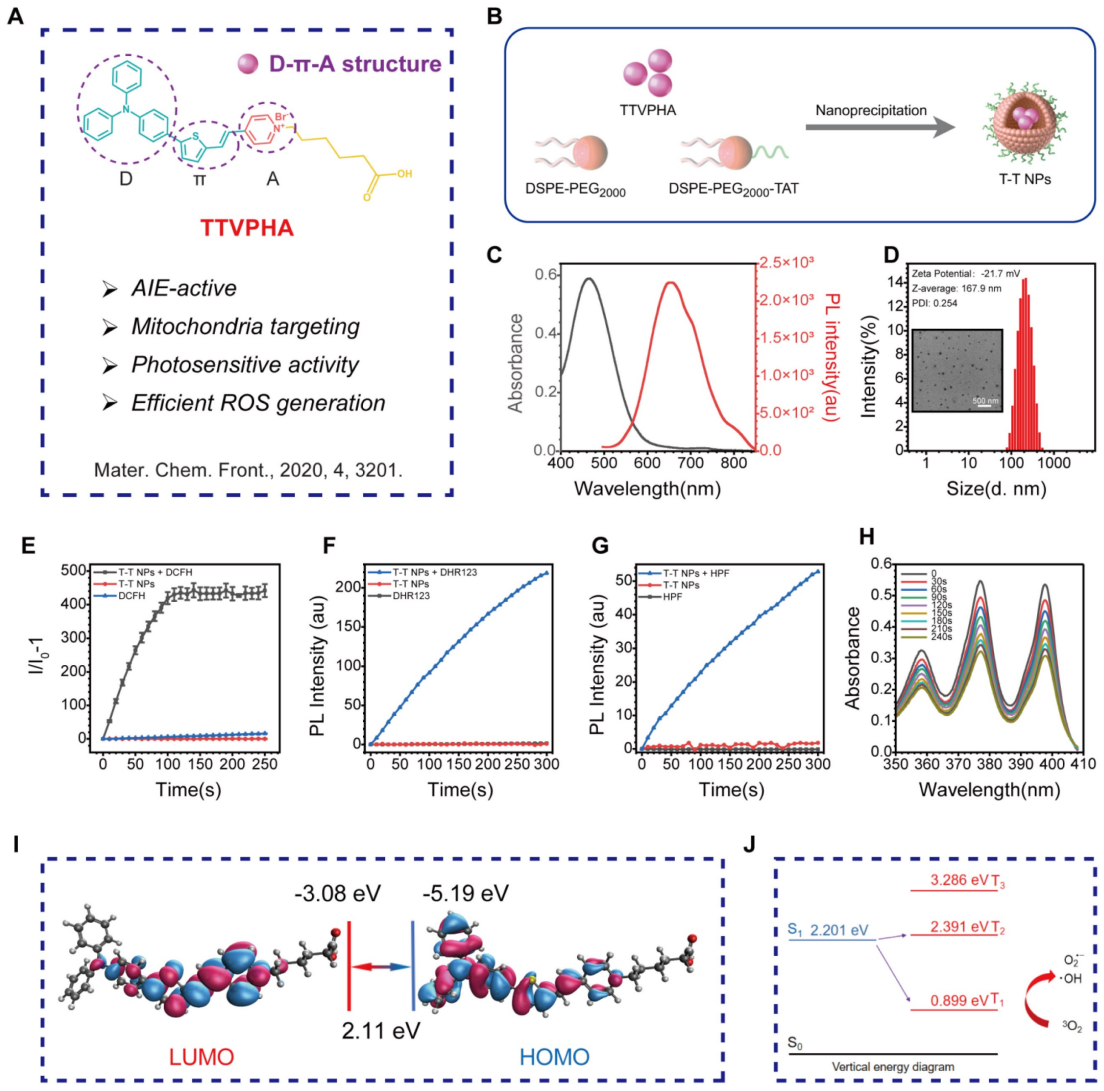
**Preparation and physicochemical characterizations of T-T NPs. (A)** The chemical structure of AIE-active PS TTVPHA. **(B)** Schematic illustration of T-T NPs preparation. **(C)** The corresponding UV/Vis absorption spectra and fluorescence spectra of T-T NPs. **(D)** Size distributions and Zeta potential of T-T NPs were measured by DLS, and TEM image of T-T NPs. **(E-H)** The total ROS generation of T-T NPs in aqueous solution measured by the emission intensity of DCFH (10 μM), DHR123 (10 μM) and HPF (10 μM) at 530 nm, and ABDA (50 μM) at 350~410 nm after white light irradiation (20 mW·cm^-2^), to detect O_2_^•-^, •OH and ^1^O_2_ generation, respectively. **(I)** HOMO and LUMO distributions and density functional theory calculation of TTVPHA. **(J)** Energy levels of S_0_-T_1_ and S_1_-T_2_ of TTVPHA.

Mitochondria, central to cellular processes like energy metabolism, calcium homeostasis, and cell death regulation, are critical targets in organelle-focused cancer therapy. Mitochondria-targeted AIE PSs offer significant potential to enhance PDT efficacy by promoting cancer cell death and improving overall anticancer effects 29. As shown in **Figure 1A, Figure S1**, the mitochondria-targeted AIE probe TTVPHA, synthesized with a D-π-A scaffold as previously reported, was constructed from 1-(5-Carboxypentyl)-4-methylpyridin-1-ium bromide and 5-(4-(diphenylamino)phenyl)thiophene-2-carbaldehyde, known for its photosensitive property 30. TTVPHA was then encapsulated into nanoparticles (NPs) using DSPE-PEG_2000_ and DSPE-PEG_2000_-TAT via a nanoprecipitation process (**Figure 1B**). The TAT peptide on the NPs' surface significantly enhances the cellular uptake of T-T NPs 31-34.

The absorption and emission spectra of T-T NPs in aqueous solution show a maximum absorption at 464 nm and emission at 654 nm (**Figure 1C**). Dynamic light scattering (DLS) measurements and transmission electron microscopy (TEM) imaging (**Figure 1D**) confirmed that T-T NPs formed stable nanoparticles in water with a diameter of approximately 167.9 nm and a Zeta potential of -21.7 mV. ROS generation was assessed using commercial indicators. Specifically, the total ROS produced by T-T NPs under light exposure was quantified using 2',7'-dichlorodihydrofluorescein (DCFH) (**Figures 1E, Figure S2A**). To identify specific ROS types, dihydrorhodamine 123 (DHR123), hydroxyphenyl fluorescein (HPF), and singlet oxygen sensor green (ABDA) were used to detect O_2_^•-^, •OH, and ^1^O_2_, respectively. Under white light exposure, DHR123 and HPF fluorescence intensities increased (**Figures 1F-G, Figure S2B-C**), while ABDA absorbance decreased (**Figures 1H**), indicating that T-T NPs generate significant amounts of O_2_^•-^ and •OH, with minimal ^1^O_2_ production.

To understand the photophysical properties of T-T NPs, density functional theory (DFT) and time-dependent DFT (TD-DFT) calculations were performed. The HOMO electron clouds primarily reside on the electron-rich triphenylamine-thiophene unit, with slight delocalization on the vinyl unit, while the LUMO is primarily localized on the pyridinium moiety. This illustrates a large HOMO-LUMO orbital separation and typical donor-acceptor characteristics 35. As shown in **Figure 1I**, the HOMO-LUMO separation facilitates intersystem crossing (ISC) by reducing the ΔE_ST_ value, with energy gaps ΔE_S1-T2_ and ΔE_S1-T1_ calculated at -0.190 eV and 1.302 eV, respectively. The smaller S_1_-T_2_ energy gap suggests an efficient ISC from S_1_ to T_2_, with internal conversion from T_2_ to T_1_ further supporting ROS generation. Additionally, the triplet-ground state energy gap (ΔE_T1-S0_) of TTVPHA was calculated to be 0.899 eV, lower than the ^3^O_2_ and ^1^O_2_ energy gap (1.61 eV), indicating that T-T NPs are efficient in generating type I ROS (O_2_^•-^ and •OH) (**Figure 1J**). These results demonstrate that T-T NPs can generate ROS through both type I and type II photosensitized oxidation, which is advantageous for PDT in hypoxic tumors by mitigating oxygen depletion during treatment.

### Cellular uptake of T-T NPs* in vitro*

**Figure 2:**
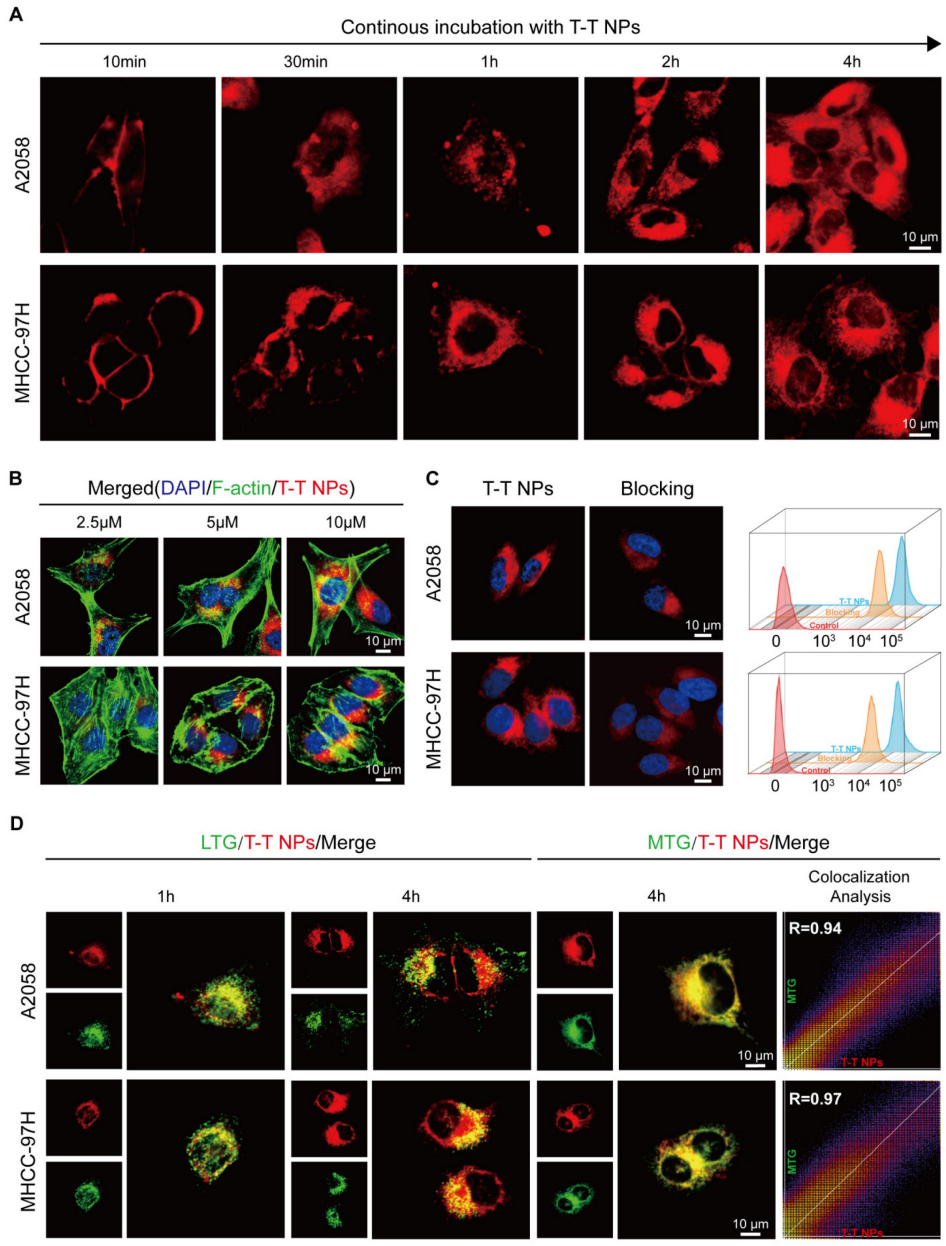
** The cellular uptake and organelle-targeting of T-T NPs in A2058 cells and MHCC 97H cells. (A)** Dynamic fluorescence images of T-T NPs accumulation in cells with different incubation time monitored by confocal laser scanning microscopy (CLSM). **(B)** Cellular uptake of T-T NPs in cells with different concentrations monitored by CLSM. **(C)** Confocal images and Flow cytometry analysis of cells after incubation with T-T NPs for 4 h. Blocking group was pretreated by genistein for 2 h. **(D)** Co-localization imaging and quantitation analysis of A2058 cells and MHCC 97H cells stained with T-T NPs, LysoTracker Green (LTG) and MitoTracker Green (MTG) observed by CLSM.

Confocal laser scanning microscopy (CLSM) was employed for real-time, *in situ* monitoring of T-T NP cell entry in human melanoma A2058 and human hepatocellular carcinoma MHCC 97H cells. As shown in **Figure 2A, Figure S3**, strong red fluorescence from T-T NPs was initially observed on the cell membrane of both A2058 and MHCC 97H cells after 10 min of incubation. With continued incubation up to 4 h, red fluorescence increasingly localized in the cytoplasm. This progressive increase in fluorescence intensity corresponded with the concentration of T-T NPs, as confirmed by co-staining with the blue-emissive nuclei dye DAPI and the green-emissive cytoskeleton dye F-actin in **Figures 2B, Figure S4-5**. To assess the impact of TAT on T-T NPs uptake, tumor cells were pre-treated with genistein, a TAT inhibitor 36, which significantly reduced T-T NPs internalization, as seen in both CLSM images and flow cytometry (FCM) analysis in **Figure 2C**. Further experiments were conducted to investigate the organelle-targeting capability of T-T NPs through co-staining with LysoTracker Green (LTG) and MitoTracker Green (MTG). As illustrated in **Figure 2D**, red fluorescence from T-T NPs initially showed high co-localization with LTG after 1 h of incubation. However, with additional incubation to 4 h, the co-localization ratio with LTG decreased significantly from 0.88 to 0.51 in A2058 cells and from 0.89 to 0.59 in MHCC 97H cells (**Figure S6**). Concurrently, high Pearson's correlation coefficients of 0.94 and 0.97 with MTG were observed in A2058 and MHCC 97H cells, respectively, indicating strong mitochondrial targeting. These results suggest that TTVPHA gradually escapes from T-T NPs and accumulates in mitochondria, likely due to its positive charge and delocalization properties. This mitochondrial targeting enhances its potential in anticancer therapy and provides a basis for further exploration of the mechanisms through which T-T NPs exert tumor cell damage.

### Photodynamic therapeutic performance *in vitro*

**Figure 3:**
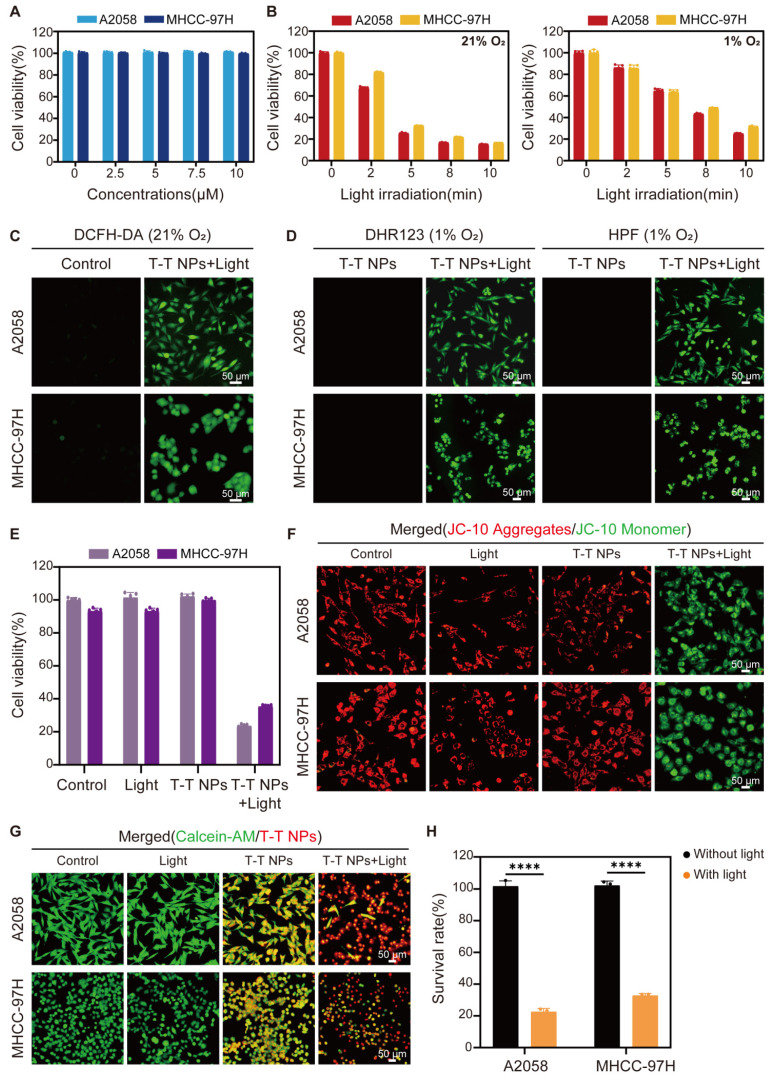
***In vitro* Photodynamic therapeutic performance of T-T NPs on A2058 and MHCC 97H cells. (A)** Cytotoxicity of A2058 and MHCC 97H cells treated with varying concentrations of T-T NPs without light exposure under normoxic conditions, assessed by CCK8 assay. **(B)** Time-dependent phototoxicity of T-T NPs under normoxic and hypoxic conditions. **(C)** Intracellular ROS generation observed by fluorescence microscopy in T-T NPs-treated cells after light exposure, using DCFH-DA (20 μM) as a probe under normoxia. **(D)** Detection of intracellular O_2_^•-^ and •OH using DHR123 (20 μM) and HPF (20 μM) indicators in the presence of T-T NPs, with or without light exposure under hypoxia. **(E)** Cell viability of A2058 and MHCC 97H cells subjected to different treatments, measured by CCK8 assay. **(F)** Mitochondrial membrane potential (∆Ψ_m_) in A2058 and MHCC 97H cells following various treatments, stained with the JC-10 probe. **(G)** Fluorescence imaging and **(H)** quantitative analysis of live cell staining using Calcein-AM (2 μM) for A2058 and MHCC 97H cells under different treatments. ****p < 0.0001. Light exposure = 300 mW·cm⁻², [TTVPHA] = 5 μM.

After confirming the efficient uptake of T-T NPs by cancer cells and their mitochondria-targeted accumulation of TTVPHA, we evaluated the photodynamic therapeutic efficacy of T-T NPs. In the dark, T-T NPs showed excellent biocompatibility, maintaining cell viability close to 100% even at a high concentration of 10 μM TTVPHA under both normoxic (21% O₂) and hypoxic (1% O₂) conditions (**Figure 3A, Figure S7**). In contrast, the survival rates of A2058 and MHCC 97H cells incubated with T-T NPs containing 5 μM TTVPHA gradually decreased with extended light exposure from 0 to 10 min (**Figure 3B**). For comparison, the control groups with only light or T-T NPs in the dark showed no decrease in cell viability (**Figure 3E**). The robust ROS generation capability of T-T NPs under light irradiation was confirmed by the ROS indicator DCFH-DA under normoxic conditions (**Figure 3C, Figure S8**). Specific ROS fluorescent probes, including DHR123 for O_2_^•-^ and HPF for •OH, were used to verify TTVPHA's type I photodynamic ability. As shown in **Figure 3D, Figure S9**, under hypoxia, the fluorescence intensities of DHR123 and HPF sharply increased in the presence of T-T NPs upon light exposure, strongly indicating that TTVPHA retains its type I photosensitizer properties in cells, producing ROS independently of oxygen levels—a crucial advantage for enhanced tumor-killing efficacy. Due to TTVPHA's mitochondrial targeting, we further investigated whether PDT-induced ROS could damage mitochondria to enhance tumor cell death. Using the JC-10 probe to indicate mitochondrial membrane potential, the PDT group showed primarily green fluorescence from JC-10 monomers, indicating significant mitochondrial membrane potential depolarization (Δψm) compared to other groups (**Figure 3F, Figure S10**). Subsequent live-cell staining with Calcein-AM under identical light conditions revealed that only 20-30% of cells remained viable in the PDT group (**Figure 3G-H**), corroborating the CCK8 assay results. Overall, these findings demonstrate that T-T NPs-mediated PDT generates substantial ROS levels under both normoxic and hypoxic conditions, resulting in mitochondrial damage and potent cytotoxicity against tumor cells. These results underscore the promising therapeutic potential of T-T NPs for anticancer treatment.

### Potential therapeutic mechanism of T-T NPs-mediated PDT

**Figure 4:**
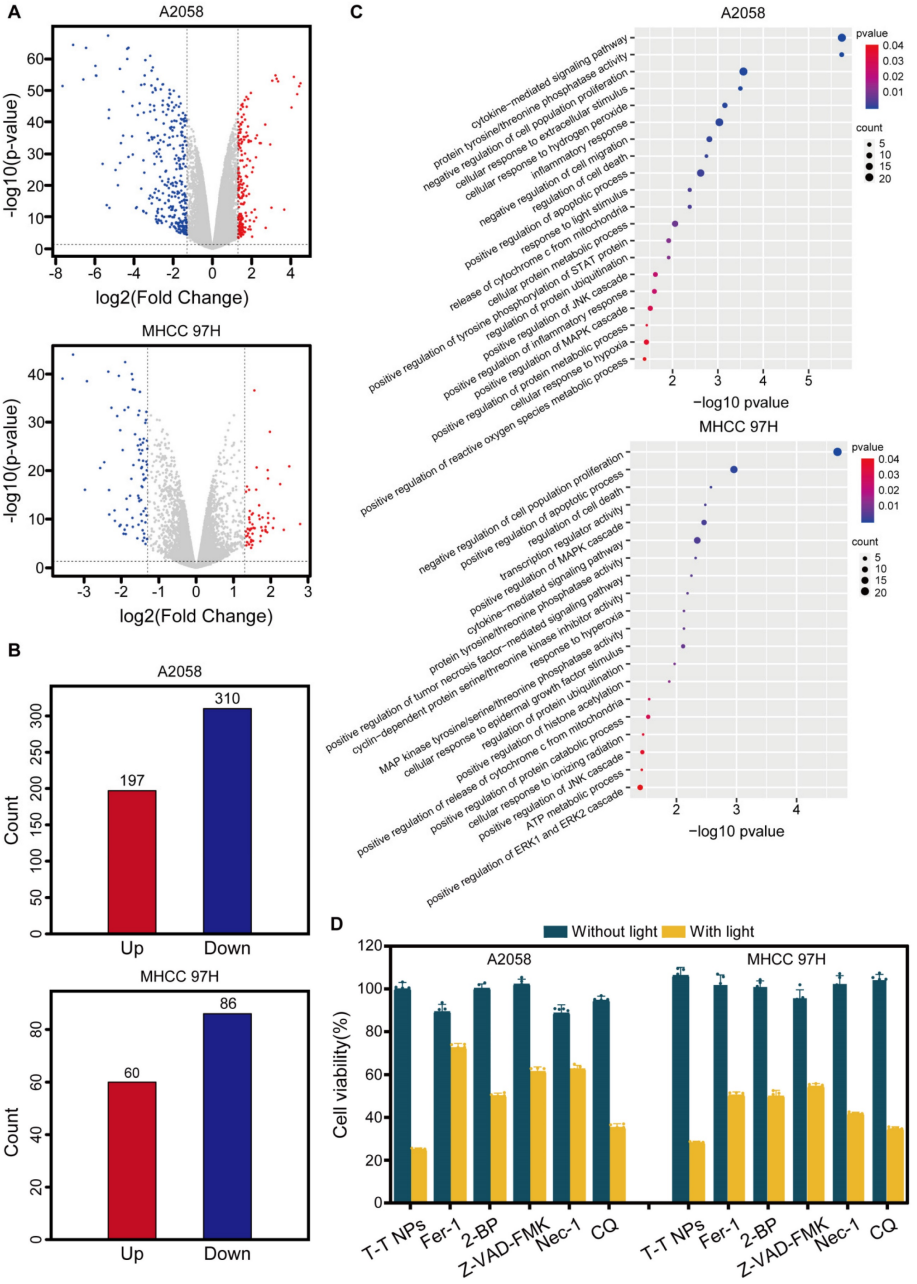
** Investigation of potential cell death mechanism in T-T NPs-mediated PDT on A2058 and MHCC 97H cells. (A)** Volcano plot of DEGs in A2058 and MHCC 97H cells under control and PDT conditions. **(B)** Histogram of DEG counts in the control versus PDT groups. **(C)** GO pathway enrichment analysis of altered genes in the PDT group compared to the control group.** (D)** Cell viability of A2058 and MHCC 97H cells treated with T-T NPs with or without light exposure in combination with various inhibitors: ferroptosis inhibitor ferrostatin-1 (Fer-1, 50 µM), pyroptosis inhibitor 2-bromohexadecanoic acid (2-BP, 5 µM), apoptosis inhibitor Z-VAD-FMK (100 µM), necroptosis inhibitor necrostatin-1 (Nec-1, 50 µM), and autophagy inhibitor chloroquine (CQ, 20 µM).

To elucidate the mechanisms underlying T-T NPs-mediated PDT, whole-transcriptome analysis was conducted, identifying 507 and 146 differentially expressed genes (DEGs) (|log₂(Fold Change)| ≥ 1, p < 0.05) in A2058 and MHCC 97H cells, respectively (**Figure 4B**). Volcano plots depict the upregulated and downregulated genes (**Figure 4A**). Gene Ontology (GO) pathway enrichment analysis of DEGs in both cell lines (**Figure 4C**) revealed significant enrichment in biological processes (BP), with DEGs associated with multiple functions, including negative regulation of cell proliferation and positive regulation of apoptosis and metabolic processes. These DEGs are also linked to protein ubiquitination regulation and pathways such as the JNK/MAPK cascade 37, histone acetylation 38-39, and tyrosine/threonine phosphatase activity 40. These pathways are known to participate in various cell death mechanisms, including apoptosis, pyroptosis, ferroptosis and necroptosis. Based on these observations, we hypothesize that PDT with T-T NPs induces multiple cancer cell death pathways. To test this hypothesis, cancer cells were pretreated with inhibitors specific to various cell death pathways before T-T NPs treatment, including Z-VAD-FMK (apoptosis inhibitor), ferrostatin-1 (ferroptosis inhibitor), necrostatin-1 (necroptosis inhibitor), chloroquine (autophagy inhibitor), and 2-bromohexadecanoic acid (pyroptosis inhibitor). All inhibitors increased cell survival and mitigated the therapeutic effects of T-T NPs under light exposure (**Figure 4D**). These findings indicate that mitochondria-targeted PDT can trigger multiple cell death pathways, warranting further investigation into the detailed therapeutic mechanisms.

### PANoptosis induced by T-T NPs-mediated PDT

PANoptosis, a form of programmed cell death (PCD), integrates features of apoptosis, necroptosis, and pyroptosis, representing a complex and combined mode of cell death that engages multiple signaling pathways and various effector mechanisms 41-42. The accumulation of ROS activates several apoptosis-related signaling pathways, including the mitochondrial apoptosis pathway (involving Bcl-2 family proteins), caspase cascades, and transcription factors like p53, all contributing to apoptosis induction. Studies indicate that ROS can downregulate anti-apoptotic proteins (e.g., Bcl-2), triggering apoptosis by activating pro-apoptotic proteins like BAX 43. Activated BAX disrupts the mitochondrial outer membrane, releasing mitochondrial proteins into the cytosol and activating caspase family proteins to initiate apoptosis 44.

**Figure 5:**
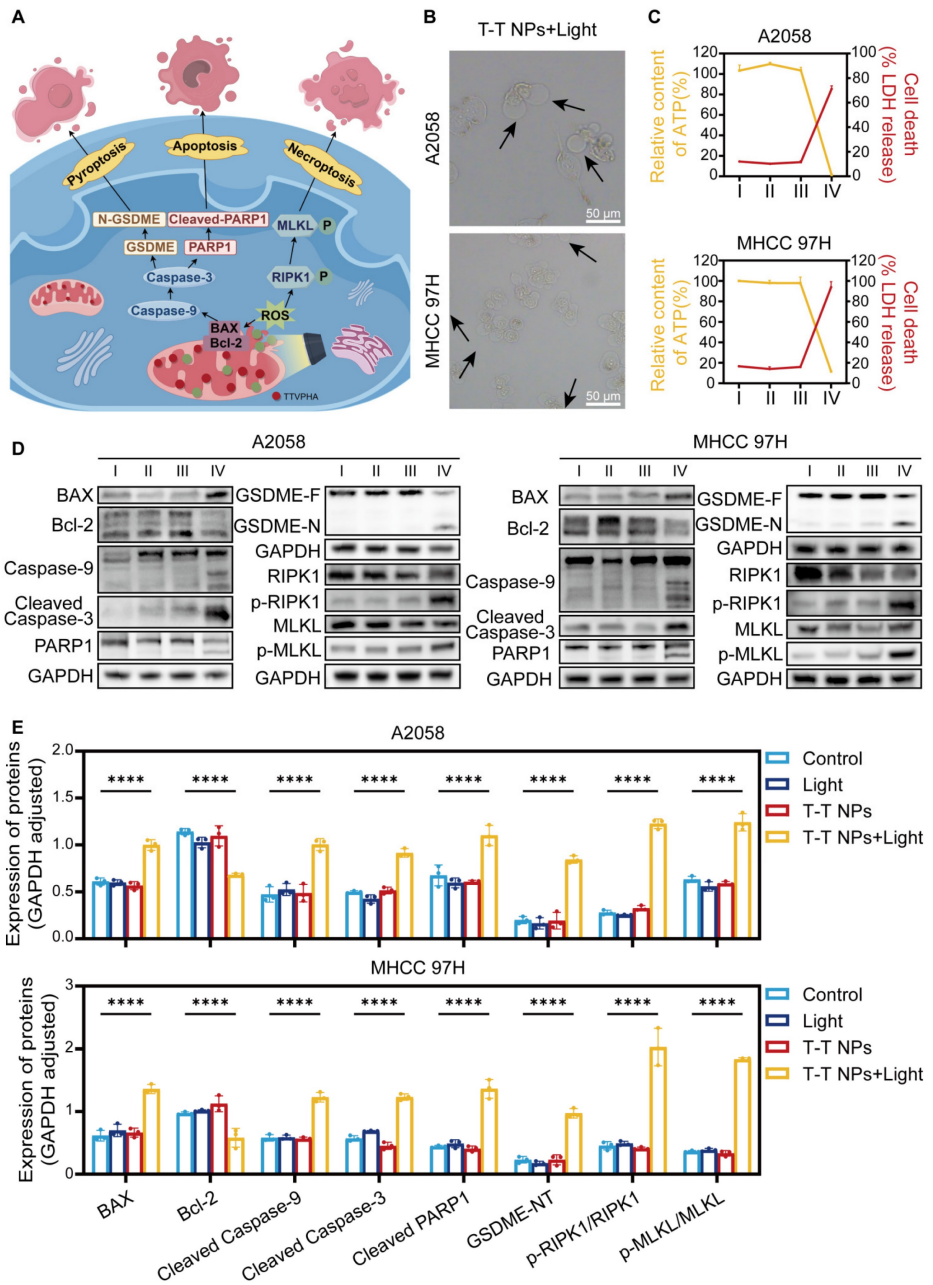
** Characterization of PANoptosis induced by T-T NPs in A2058 and MHCC 97H cells. (A)** Schematic of the PANoptosis mechanism induced by T-T NPs. **(B)** Representative images showing changes in cell morphology in A2058 and MHCC 97H cells following PDT treatment. **(C)** Measurement of extracellular lactate dehydrogenase (LDH) and intracellular adenosine triphosphate (ATP) in A2058 and MHCC 97H cells under different treatment conditions. **(D)** Western blot analysis of key proteins associated with PANoptosis in A2058 and MHCC 97H cells (I: Control, II: Light, III: T-T NPs, IV: T-T NPs + Light). **(E)** Semi-quantitative analysis of PANoptosis protein expression in A2058 and MHCC 97H cells under different treatments. ****p < 0.0001.

Caspase-9 then cleaves caspase-3, which induces poly (ADP-ribose) polymerase 1 (PARP1) activation, leading to apoptosis 45, and also cleaves gasdermin E (GSDME), releasing the GSDME-N domain and causing vesicle formation, thereby inducing pyroptosis 46. Additionally, high mitochondrial ROS levels promote receptor-interacting serine/threonine-protein kinase 1 (RIPK1) phosphorylation, which further phosphorylates mixed lineage kinase domain-like pseudokinase (MLKL), ultimately disrupting the plasma membrane, releasing cellular contents, and promoting necroptosis 47. Based on these pathways, we hypothesize that T-T NPs-mediated PDT could induce PANoptosis in tumor cells, as illustrated in **Figure 5A**.

As shown in **Figure 5B, Figure S11**, following PDT treatment, visible membrane bubbling was observed in both A2058 and MHCC 97H cells, a characteristic feature of pyroptosis, while no distinct morphological changes appeared in the other treatment groups. This suggests that T-T NPs-mediated PDT effectively induces pyroptosis. Pyroptotic markers, including lactate dehydrogenase (LDH) and adenosine triphosphate (ATP), were then quantitatively assessed. In the T-T NPs + Light group, intracellular ATP levels significantly decreased, while extracellular LDH levels increased markedly compared to the control groups (Control, Light, T-T NPs), further indicating pyroptosis induction by T-T NPs-mediated PDT (**Figure 5C**). Western blot analysis (**Figure 5D-E**) revealed key proteins involved in pyroptosis, apoptosis, and necroptosis. In the T-T NPs + Light group, there was a notable decrease in the anti-apoptotic protein Bcl-2, accompanied by a significant upregulation of the pro-apoptotic protein BAX. The increase in BAX led to the activation of caspase-9, confirming the engagement of the intrinsic apoptotic pathway. Cleaved caspase-3, a central executioner in apoptosis, was also detected, indicating the activation of the apoptotic cascade 45. Caspase-3 cleaves GSDME, generating an N-terminal fragment that forms pores in the plasma membrane, resulting in pyroptosis 46. The detection of cleaved GSDME in cells treated with PDT supports the simultaneous occurrence of pyroptosis and apoptosis. Additionally, cleaved caspase-3 further activated PARP1, reflecting apoptosis involvement. Phosphorylation of necroptosis-related proteins RIPK1 and MLKL was also observed, validating that necroptosis was triggered by PDT treatment. Altogether, these findings strongly support the induction of PANoptosis by T-T NPs-mediated PDT both in A2058 and MHCC 97H cells, revealing the complex activation of apoptosis, pyroptosis, and necroptosis. This multimodal mechanism offers a potentially comprehensive approach for effectively targeting cancer cells.

### Ferroptosis induced by T-T NPs-mediated PDT

**Figure 6:**
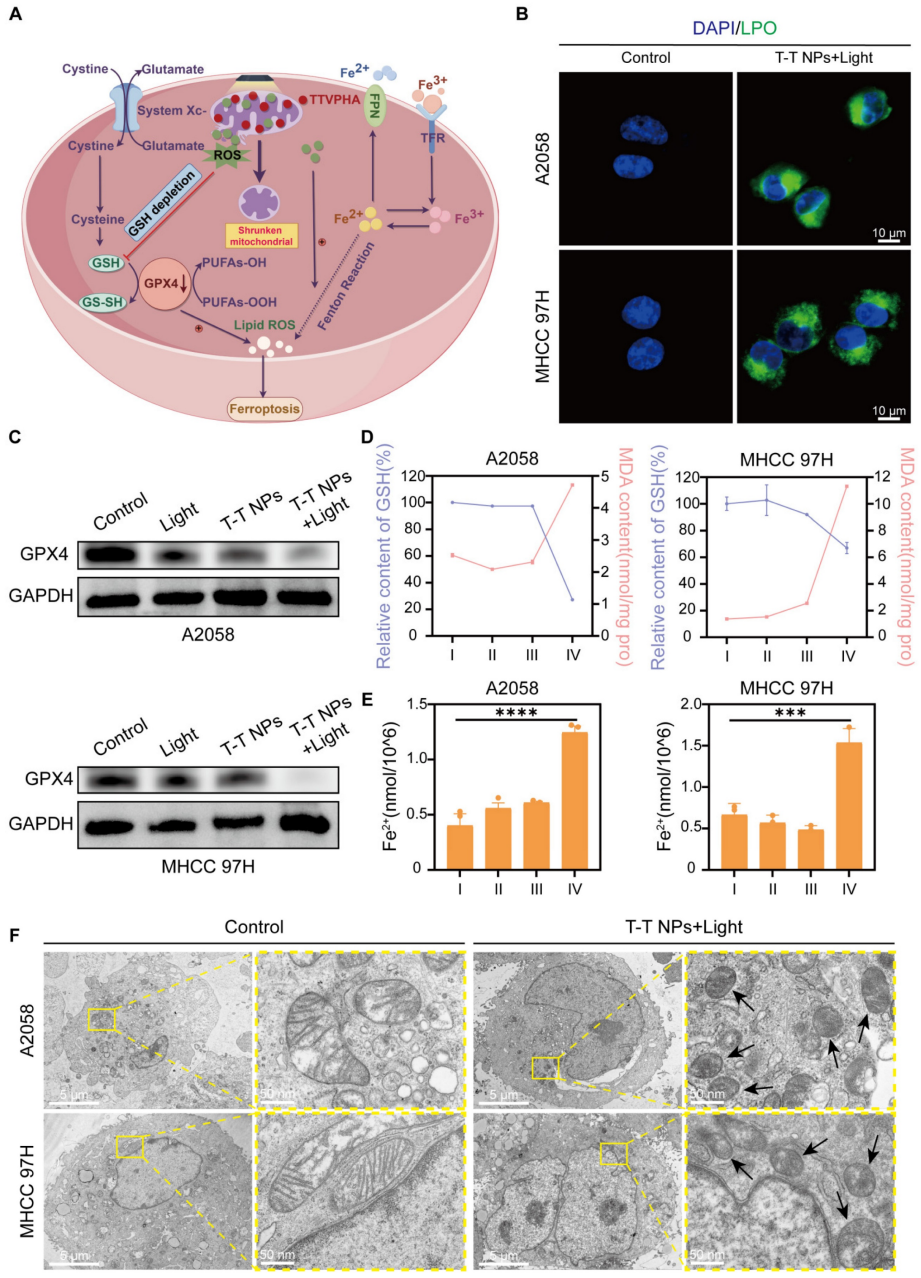
** Characterization of ferroptosis triggered by T-T NPs in A2058 and MHCC 97H cells. (A)** Schematic representation of the mechanism of ferroptosis induced by T-T NPs. **(B)** Confocal imaging of lipid peroxidation in A2058 and MHCC 97H cells stained with C11-BODIPY (581/591) dye following PDT with T-T NPs. **(C)** Western blot analysis of glutathione peroxidase 4 (GPX4) expression in A2058 and MHCC 97H cells under different treatment conditions. **(D)** Quantification of glutathione (GSH) and malondialdehyde (MDA) levels, and **(E)** evaluation of Fe²⁺ levels in A2058 and MHCC 97H cells across different formulations (I: Control; II: Light; III: T-T NPs; IV: T-T NPs + Light). ***p < 0.001; ****p < 0.0001. **(F)** Transmission electron microscopy (TEM) images of mitochondria in A2058 and MHCC 97H cells after PDT with T-T NPs.

Ferroptosis is a distinct type of regulated cell death, unlike apoptosis, necrosis, and autophagy. It is marked by iron-dependent lipid peroxide accumulation, ultimately leading to cell death, and is tightly controlled by the balance between pro-oxidation and anti-oxidation processes 48-50. Excessive ROS production can deplete intracellular glutathione (GSH) and downregulate glutathione peroxidase 4 (GPX4), resulting in lipid peroxidation (LPO) and triggering ferroptosis 14, 51. ROS can also interact with ferrous iron through the Fenton reaction, generating toxic hydroxyl radicals that further promote LPO and ferroptosis 52. Thus, we hypothesize that T-T NPs-mediated PDT could induce ferroptosis in tumor cells, as illustrated in **Figure 6A**. Our prior CCK8 results confirmed that the ferroptosis inhibitor ferrostatin-1 (Fer-1) could mitigate the cytotoxic effects of T-T NPs-mediated PDT on A2058 and MHCC 97H cells (**Figure 4D**). LPO, a key biomarker of ferroptosis, was quantified using the fluorescent indicator C11-BODIPY (581/591) dye, and a significantly stronger green fluorescence was observed in the T-T NPs + Light group compared to other groups (**Figure 6B, Figure S12**). Additionally, **Figure 6C, Figure S13** show markedly reduced expression of GPX4 protein in A2058 and MHCC 97H cells treated with T-T NPs + Light, as revealed by Western blot analysis. Malondialdehyde (MDA), a byproduct of lipid oxidation, serves as an indicator of lipid oxidation under oxidative stress. The elevated MDA levels, along with a substantial reduction in GSH levels and a sharp increase in cellular ferrous iron (**Figures 6D-E**), further support the ability of T-T NPs-mediated PDT to induce strong ferroptosis. Transmission electron microscopy (TEM) was used to examine mitochondrial morphology in cells under different treatments. TEM images revealed that mitochondria in tumor cells treated with T-T NPs + Light displayed classic ferroptotic morphology 48-49, including notable shrinkage and increased mitochondrial membrane density, distinctly different from normal mitochondria (**Figure 6F**). Collectively, these findings demonstrate that T-T NPs can efficaciously induce ferroptosis in tumor cells through PDT.

### *In vivo* biosafety, tumor imaging and distribution

**Figure 7:**
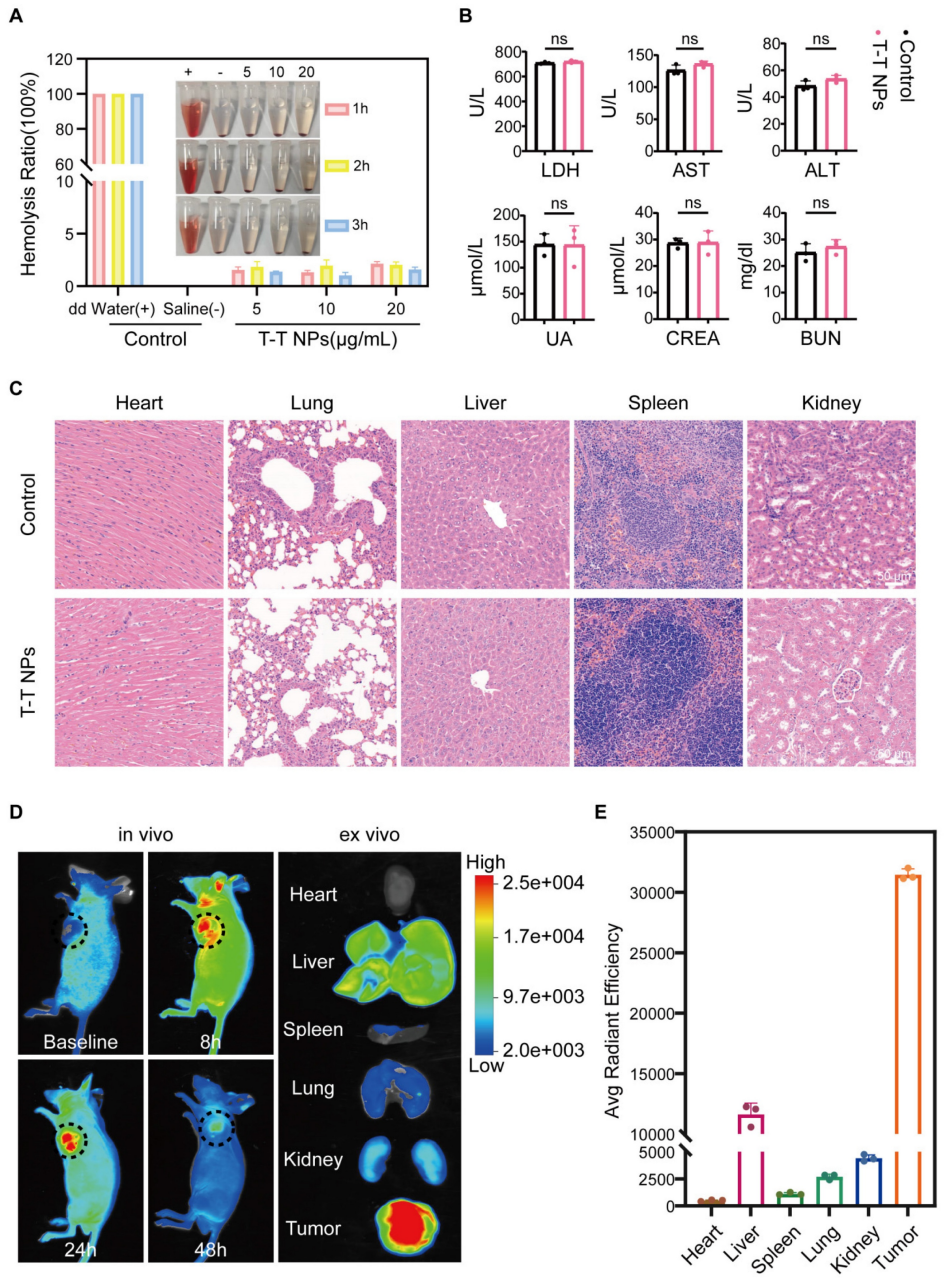
** Biocompatibility and biodistribution of T-T NPs *in vivo*. (A)** Hemolysis assay of mouse red blood cells exposed to T-T NPs at varying concentrations over a set period. Inset: Optical image showing hemolysis extent after sample centrifugation. **(B)** Blood biochemical analysis for liver function (LDH, AST, ALT) and renal function (UA, CREA, BUN) in mice following intravenous injection of T-T NPs (30 mg/kg). Data are shown as mean ± SD (n = 3 per group), with no statistical differences observed (ns). **(C)** H&E staining of major organs following intravenous injection of T-T NPs (30 mg/kg) for histopathological examination. **(D)** *In vivo* dynamic fluorescence imaging of A2058 tumor-bearing mice at various time points post-intravenous injection of T-T NPs (30 mg/kg).* Ex vivo* fluorescence imaging of tumors and major organs harvested at 24 h post-injection of T-T NPs (30 mg/kg). **(E)** Quantitative *ex vivo* analysis of fluorescence in various organs and tumor tissue from mice treated with T-T NPs, 24 h post-injection.

To evaluate the *in vivo* antitumor potential of T-T NPs-mediated PDT, we first assessed their biosafety and imaging capabilities. Hemocompatibility assays were conducted to evaluate the biocompatibility of T-T NPs for *in vivo* use. As shown in **Figure 7A**, T-T NPs at various concentrations caused no significant hemolysis, similar to the saline-treated negative control group, while distilled water (positive control) led to substantial hemolysis. These results suggest that T-T NPs are hemocompatible and suitable for intravenous administration. For systemic toxicity evaluation, 125 μL of T-T NPs (4 mg/mL) or PBS was administered intravenously to two groups of healthy nude mice. Seven days post-injection, blood samples were collected to analyze markers of liver and kidney function, including LDH, Aspartate Aminotransferase (AST), Alanine Aminotransferase (ALT), Blood Urea Nitrogen (BUN), Creatinine (CREA), and Uric Acid (UA). Major organs (heart, liver, spleen, lung, and kidney) were harvested for histological analysis via hematoxylin and eosin (H&E) staining. As shown in **Figure 7B**, no significant differences were observed between the T-T NPs and PBS groups for these markers, and histological analysis (**Figure 7C**) revealed no pathological changes in the T-T NPs group compared to the PBS group. Together, these results indicate that T-T NPs exhibit excellent biosafety, supporting their use in *in vivo* antitumor applications. The biodistribution and tumor-targeted imaging of T-T NPs were assessed using non-invasive fluorescence imaging in an A2058 tumor-bearing mouse model. *In vivo* imaging showed peak fluorescence intensity at 24 h post-injection, suggesting gradual accumulation of T-T NPs in the tumor (**Figure 7D**). To further explore tumor-specific accumulation, *ex vivo* imaging was conducted 24 h post-injection. As shown in **Figures 7D-E**, fluorescence intensity was significantly higher in the tumor compared to major organs, indicating selective accumulation of T-T NPs in tumor tissue.

### Anti-tumour effects of T-T NPs-mediated PDT *in vivo*

**Figure 8:**
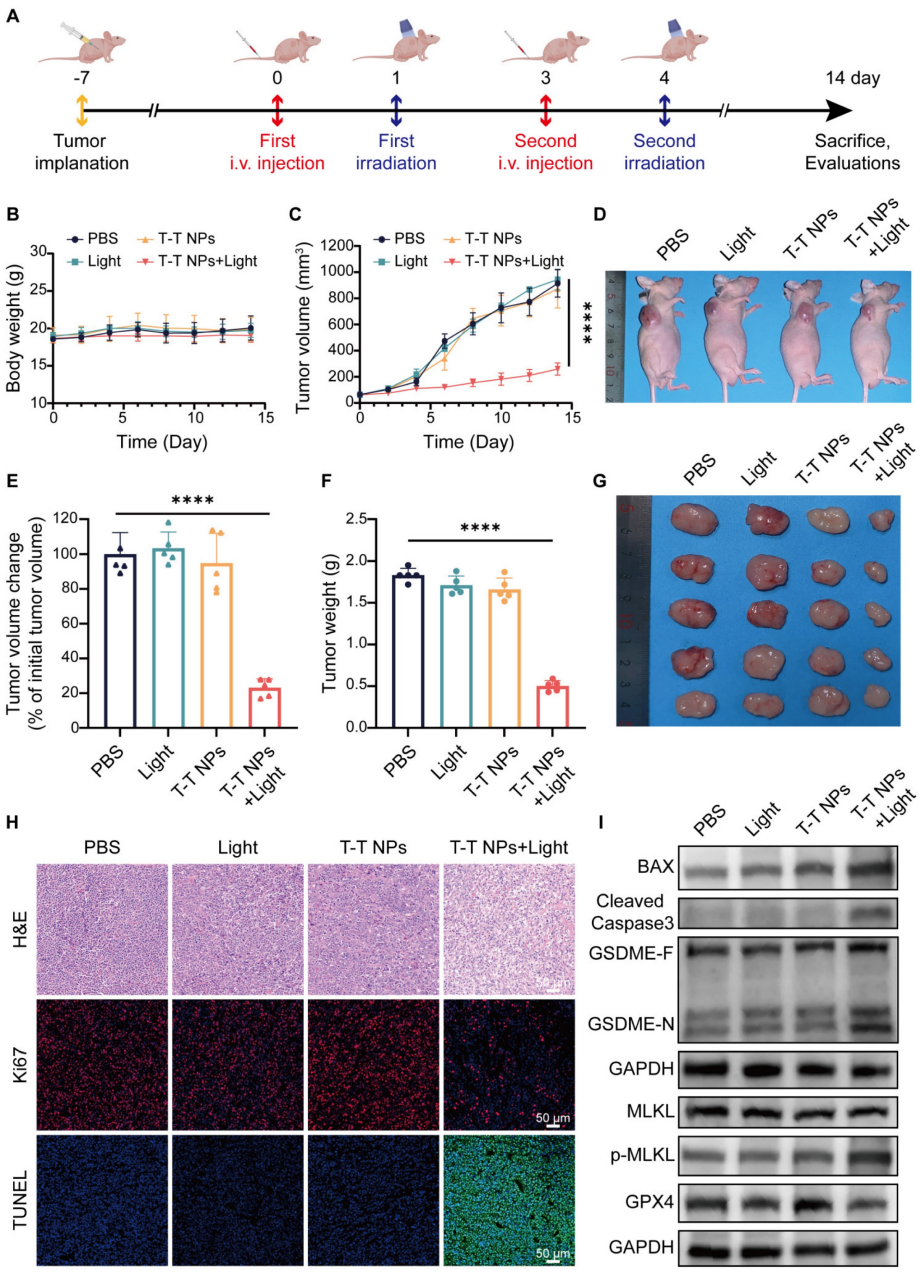
***In vivo* antitumor efficacy and mechanism of T-T NPs-mediated PDT. (A)** Schematic diagram of the experimental design for *in vivo* tumor therapy (n = 5 per group). **(B)** Body weight curves of mice over 14 days following different treatments. **(C)** Tumor volume growth curves, **(D)** representative photographs, and **(E)** semi-quantitative analysis of relative tumor volume changes in tumor-bearing mice across treatment groups. **(F)** Tumor weights and **(G)** photographs of excised tumors from mice at the end of different treatments. **(H)** H&E, Ki67, and TUNEL staining of tumor slices on day 14 post-treatment. **(I)** Western blot analysis of key protein expression in tumor tissue after different treatments. ****p < 0.0001.

To assess the antitumor efficacy of T-T NPs-mediated PDT *in vivo*, an A2058 tumor-bearing mouse model was established (**Figure 8A**). As shown in **Figure 8B**, body weight remained stable across all treatment groups, indicating excellent *in vivo* biocompatibility of T-T NPs. Furthermore, PDT-treated mice exhibited obvious tumor growth inhibition compared to other groups (**Figure 8C**). Neither the T-T NPs-only nor the light-only groups showed notable tumor inhibition, similar to the control group treated with PBS. By the end of the treatment period, the relative tumor volume in the PDT group was approximately 20% of the initial size, markedly lower than in other groups (**Figure 8E**). Representative images of treated mice display tumor sizes across different groups (**Figure 8D**). Tumors were excised, weighed, and imaged post-treatment, revealing that the T-T NPs + Light group had significantly smaller tumors and a tumor inhibition rate of approximately 72.5% (**Figures 8F-G**). Histological analyses, including H&E staining, Ki67 immunostaining, and TUNEL assays, were performed on tumor tissues. As shown in Figure 8H, the T-T NPs + Light group exhibited extensive nuclear loss, reduced Ki67 expression, and increased apoptosis (**Figure S14**), underscoring the potent antitumor effect of T-T NPs-mediated PDT *in vivo*.

To investigate whether T-T NPs-mediated PDT could induce PANoptosis and ferroptosis *in vivo*, Western blot analysis was conducted on key proteins in tumor tissues (**Figure 8I, Figure S15**). The T-T NPs + Light group showed elevated levels of pro-apoptotic protein BAX and activated caspase-3, confirming notable apoptosis in tumor tissues. The distinct cleavage of GSDME, a pyroptosis marker, suggests that T-T NPs-mediated PDT also induces pyroptosis *in vivo*. Furthermore, phosphorylated MLKL, a necroptosis marker, indicates necroptosis activation in PDT-treated tumor tissue. The co-occurrence of these cell death forms implies that PDT can induce PANoptosis *in vivo*. The decreased expression of GPX4, a ferroptosis-related protein, further demonstrates successful induction of ferroptosis. These findings collectively validate that T-T NPs-mediated PDT can simultaneously activate PANoptosis and ferroptosis in both *in vitro* and *in vivo* settings.

## Conclusion

In summary, we successfully developed mitochondria-targeted, AIE-based PS-loaded nanoparticles (T-T NPs) modified with TAT peptides. Extensive *in vitro* and *in vivo* studies demonstrated that T-T NPs-mediated PDT effectively induces both PANoptosis and ferroptosis. This work is the first to establish a therapeutic approach that combines multiple cell death mechanisms—specifically PANoptosis and ferroptosis—within tumor cells, representing a significant advancement in PDT research. By activating a multimodal cell death network, T-T NPs achieve an enhanced antitumor effect, underscoring the potential of this approach to improve therapeutic outcomes. This multimodal strategy not only maximizes tumor cell elimination but also reduces the likelihood of resistance, a frequent obstacle in cancer treatment. The findings here offer critical insights for designing novel PSs and exploring the mechanisms underlying the antitumor effects of PDT. Future research will aim to optimize delivery efficiency and specificity, investigate synergistic combinations, elucidate molecular pathways, and advance clinical translation. By addressing these objectives, this research seeks to propel PDT development and contribute to more effective, personalized cancer treatments. The multimodal cell death network induced by T-T NPs represents a promising new approach in cancer therapy, offering the potential for improved patient outcomes and a deeper understanding of tumor cell death mechanisms.

## Materials and Methods

### Materials and Instruments

TTVPHA was selected from ASBase as an AIE-characteristic PS (https://www.asbase.cn/Search/info.html?id=1901), and synthesized according to our previous report 1. DSPE-PEG_2000_-TAT and DSPE-PEG_2000_ were purchased from Xi'an Ruixi Biological Technology Co., Ltd (Xian, China). The human melanoma cell line A2058 was obtained from the Cell Bank of the Chinese Academy of Sciences (Shanghai, China) and the human hepatocellular carcinoma cell line MHCC 97H was obtained from Cellverse (iCell) Bioscience Technology Co., Ltd (Shanghai, China). Fetal bovine serum (FBS) and Dulbecco's modified Eagle's medium (DMEM) were purchased from Gibco (Grand Island, USA). Cell Counting Kit-8 (CCK8), 2',7'-Dichlorofluorescin diacetate (DCFH-DA) and JC-10 were obtained from HYCEZMBIO (Wuhan, China). Dihydrorhodamine 123 (DHR 123) was purchased from GlpBio Technology (GC30581, USA). Hydroxyphenyl Fluorescein (HPF) was obtained from MedChemExpress (MCE) (HY-111330, Shanghai, China). 9.10-Anthracenediyl-bis(methylene)dimalonic Acid (ABDA) was obtained from Maokang Technology (MK4822-50MG, Shanghai, China). C11-BODIPY (581/591) dye and PVDF transfer membranes were purchased from Thermo Fisher (Waltham, MA, USA). MitoTracker Green (MTG), Lyso-Tracker Green (LTG), DAPI, LDH release assay kit, enhanced ATP assay kit, Calcein-AM/PI dual staining kit, GSH assay kit, MDA assay kit, BCA protein assay kit, general protease inhibitor cocktail, general phosphatase inhibitor cocktail and nonfat powdered milk were purchased from Beyotime (Jiangsu, China). ECL substrate, RIPA lysis buffer, 4% paraformaldehyde, 2.5% glutaraldehyde buffer and DMSO were purchased from Biosharp (Hefei, China). GAPDH, BAX, Bcl-2, Caspase 9, PARP1, RIPK1, phospho-RIPK1, MLKL, GPX4, and HRP-labeled anti-rabbit/mouse secondary antibodies were purchased from Proteintech (Wuhan, China). Cleaved caspase-3 was purchased from Cell Signaling Technology (Danvers, MA, USA). GSDME was purchased from Abcam (Cambridge, MA, USA). Phospho-MLKL was purchased from ABclonal Technology (Wuhan, China). RIPA buffer, TBS and PBS were purchased from Servicebio Technology Co., Ltd. (Wuhan, China). The 5× sample loading buffer, protein ladder and 12.5% gel kit were purchased from Epizyme (Shanghai, China). Cell Ferrous Iron Colorimetric Assay Kit was purchased from Elabscience Biotechnology Co., Ltd. (Wuhan, China). All biological inhibitors were obtained from MedChemExpress (MCE) (Shanghai, China). UV‒vis absorption spectra was measured using a Shimadzu UV-2600 spectrophotometer and Photoluminescence (PL) spectra was recorded using a Horiba Fluoromax-4 spectrofluorometer.

### Preparation and characteristics of T-T NPs

1-(5-Carboxypentyl)-4-methylpyridin-1-ium bromide (72 mg, 0.25 mmol), 5-(4-(diphenylamino)phenyl)thiophene-2-carbaldehyde (80 mg, 0.25 mmol) and piperidine (200 μL) were mixed with ethanol (10 mL). The reaction mixture was heated and refluxed at 79 °C for 2 h. After cooling to room temperature, the solvent was removed under reduced pressure. The crude product was purified using silica-gel chromatography with CH_2_Cl_2_/CH_3_OH= 80/20 as the eluent to afford TTVPHA as a red powder with a yield of 55.8% (87 mg). ^1^H NMR (400 MHz, MeOD) δ 8.69 (d, J = 6.9 Hz, 2H), 8.05 (dd, J = 11.4, 8.1 Hz, 3H), 7.59-7.54 (m, 2H), 7.44 (d, J = 3.9 Hz, 1H), 7.37-7.27 (m, 5H), 7.13-6.98 (m, 9H), 4.47 (t, J = 7.4 Hz, 2H), 2.18 (t, J = 7.1 Hz, 2H), 2.04-1.95 (m, 2H), 1.67 (dt, J = 14.7, 7.2 Hz, 2H), 1.41 (dd, J = 15.1, 7.9 Hz, 2H) (Figure S1).

The TTVPHA (1 mg) and DSPE-PEG_2000_-TAT (3 mg) with DSPE-PEG_2000_ (mass ratio = 1:1) were dissolved together in 1 mL methanol. It was slowly dripped into 9 mL dd water with a long needle in an ice bath and was sonicated (20% output power for 10 min, SCIENTZ-II D ultrasonicator) to prepare T-T NPs solution. After filtration through a 0.45 µm membrane, the solution was further concentrated by ultrafiltration centrifugation at 4 °C (5000 rpm, 30 min). The concentration of TTVPHA in the NPs was measured by comparing the absorption at 478 nm of the NPs diluted in methanol to a known concentration of TTVPHA, according to the Lambert-Beer law. Then, it was diluted with PBS to a certain concentration for subsequent experiments.

Photoluminescence (PL) spectra and UV-visible absorption spectroscopy were used to evaluate an aqueous solution of T-T NPs. The T-T NPs' dimensions and surface potential were measured by dynamic light scattering (DLS), and their nanoscopic morphology was confirmed through transmission electron microscopy (TEM). To assess the stability of the nanoparticles, the size distribution of T-T NPs was examined after varying storage times.

### Total ROS generation efficiency of T-T NPs

2',7'-dichlorodihydrofluorescein diacetate (DCFH-DA) was first dissolved in ethanol to prepare a 1 mM stock solution. Subsequently, 500 μL of this stock was mixed with 2 mL of 0.01 M NaOH and allowed to react for 30 min at room temperature in the dark. The reaction was then neutralized by adding 10 mL of PBS, resulting in a 40 μM DCFH solution, which was stored at -20 °C in the dark. For the assay, DCFH (10 μM) was combined with T-T NPs (20 μM) in PBS, and after exposure to white light, the fluorescence intensity at 525 nm, upon excitation at 488 nm, was measured to quantify ROS production.

### Type I and type II ROS generation efficiency of T-T NPs

Dihydrorhodamine 123 (DHR123) was used as an O_2_^•-^ indicator. A solution of DHR123 (10 μM) and T-T NPs (20 μM) in DMSO/PBS (1:99) was prepared, and after exposure to white light, the fluorescence increase at 528 nm upon excitation at 485 nm was measured.

For •OH detection, Hydroxyphenyl Flavin (HPF) was employed. HPF (10 μM) and T-T NPs (20 μM) were mixed in DMF/PBS (1:99), and following white light irradiation, the fluorescence enhancement at 525 nm with excitation at 490 nm was assessed.

For ^1^O_2_ detection, 9.10-Anthracenediyl-bis(methylene)dimalonic Acid (ABDA) was used. ABDA (50 μM) was mixed with T-T NPs (20 μM) in PBS containing 1% DMSO. After exposure to white light, the changes in absorbance between 350-410 nm were measured.

### Cell culture

A2058 cells and MHCC 97H cells were cultured in high-glucose Dulbecco's modified Eagle's medium (DMEM, Gibco) with 10% fetal bovine serum (FBS, Gibco) and 1% penicillin-streptomycin (P/S). The cells were all cultured in a constant temperature incubator at 37 °C with 5% CO_2_ and 90%relative humidity.

### Cell uptake and fluorescence imaging

For the detection of the intracellular distribution of T-T NPs after various incubation times, the cells were inoculated in laser confocal dishes and cultured overnight for adhesion. The culture medium was removed, and the cells were incubated with 5 μM T-T NPs for 10 min, 30 min, 1 h, 2 h and 4 h, respectively. After washing three times with PBS, the cells were observed and imaged directly using a confocal laser scanning microscope (CLSM, Nikon Corporation, Japan). For T-T NPs, λ_ex_ = 488 nm, the bandpass filter λ = 552-617 nm.

To investigate the intracellular distribution of NPs with different incubation concentrations, the cells were inoculated in laser confocal dishes overnight then incubated with 2.5, 5 and 10 μM T-T NPs for 4 h, respectviely. The blocking group was pretreated with genistein (MCE, HY-14596) for 2 h before incubating T-T NPs. After rising with PBS three times, the cells were fixed with 4% paraformaldehyde (Biosharp, BL539A) for 10 min and were stained with 10 μg/mL of Actin-Tracker Green (F-actin, Beyotime, C2201S) at 37 °C in the dark for 40 min. Immediately following three washes with PBS, the cell nucleus was labeled with 5 μg/mL DAPI (Invitrogen, 62248) for 5 min. After washing three times with PBS, the cells were subjected to imaging analysis directly using CLSM (Nikon Corporation, Japan). For DAPI, λex = 405 nm, the bandpass filter λ = 425-475 nm. For F-actin, λex = 488 nm, the bandpass filter λ = 500-530 nm. Imaging analysis was performed directly using NIS-Eliments software (Nikon, Japan).

### Quantitative analysis of T-T NPs uptake using flow cytometry

The A2058 cells and MHCC 97H cells were homogeneously inoculated in cell culture dishes and placed in a cell culture incubator overnight for adherent growth. After washing once with PBS, each dish was added with serum-free medium containing different concentrations of T-T NPs (2.5, 5 and 10 μM) incubating for 4 h. After washing three times with PBS, the cells in culture dishes were digested into flow tubes using trypsin and resuspended in 200 μL of PBS. Flow cytometry (BD Falcon, San Jose, CA, USA) was immediately used to quantitate the intracellular fluorescence intensity, and the data were analyzed using FlowJo 10.8.1. For T-T NPs, λex = 488 nm, the bandpass filter λ = 605 nm.

### Colocalization analysis between T-T NPs and mitochondrion tracker and lysosome tracker

The A2058 cells and MHCC 97H cells were homogeneously seeded in 35-mm confocal dishes, cultured overnight, and incubated with 5 μM T-T NPs for 1 h or 4 h. After three washes with PBS, each dish was incubated with Mito-Tracker Green (MTG, Beyotime, C1048) or Lyso-Tracker Green (LTG, Beyotime, C1047S) for 45 min. Real-time imaging was captured using CLSM (Nikon Corporation, Japan), and the co-localization data were analyzed using ImageJ (National Institutes of Health Free Software, USA). For MTG, λ_ex_ = 488 nm, the bandpass filter λ = 500-530 nm. For LTG, λ_ex_ = 488 nm, the bandpass filter λ = 500-530 nm.

### Cell viability assay

Cell Viability of A2058 cells and MHCC 97H cells after different treatments was detected by CCK8 assay. To assess the dark toxicity of T-T NPs, after culturing 1×10^4^ cells overnight 96-well plates, the medium containing a series of concentrations of T-T NPs (0, 2.5, 5, 7.5, or 10 μM) for 4 h under hypoxic or normoxic conditions, and after washing three times with PBS, the plates were continuously cultured for 24 h. Subsequently, the original medium was removed and the medium containing CCK8 was added and incubated for 2 h, and A450 was measured by Multimode Plate Reader (PerkinElmer Pte. Ltd., Singapore).

To assess the cytotoxicity induced by PDT under different light irradiation times, the cells in the 96-well plate were incubated with 5 μM T-T NPs for 4 h under hypoxic or normoxic conditions in the dark then exposed to blue laser irradiation (300 mW·cm^-2^) for different times (0, 2, 5, 8, and 10 min). The cells were cultured for 24 h, and the cell viability was measured according to the above description.

When CCK8 was used to detect the photodynamic effects in different treatment groups, the resulting cells were treated under the following conditions: negtive control group (without any treatment), only light group (exposed to blue laser for 5 min), only T-T NPs group (incubating with 5 μM T-T NPs for 4 h) and T-T NPs + Light (incubating with 5 μM T-T NPs for 4 h and exposed to blue laser for 5 min). The cells were suequentially cultured for 24 h, and the cell viability was measured according to the above description.

### Intracellular ROS detection

The total ROS generation ability induced by PDT was detected by the fluorescent probe DCFH-DA according to the manufacturer's instructions for the ROS detection kit (HYCEZMBIO, 040-100T). The DHR123 and HPF were used as O_2_^•-^ and •OH indicators, respectively.

For normoxic conditions, after attachment of A2058 cells and MHCC 97H cells cultured in 35-mm cell culture plates, the cells were incubated with 5 μM T-T NPs for 4 h in a 21% O₂ atmosphere. Then the DCFH-DA fluorescent probe was loaded. After incubation at 37 °C for 20 min in the dark, the medium was replaced with fresh medium, and the cells were exposed to blue light irradiation for 2 min at a power density of 300 mW·cm^-2^ then immediately imaged using a fluorescence microscope (IX71, Olympus, Tokyo, Japan). The cells incubated with Rosup solution for 30 min were treated as the positive control. For DCFH-DA, λ_ex_ = 488 nm, the bandpass filter λ = 500-550 nm.

For hypoxic condition, after attachment of A2058 cells and MHCC 97H cells cultured in 35-mm cell culture plates, the cells were incubated with 5 μM T-T NPs for 4 h in a 1% O₂ atmosphere. Then the DHR123 and HPF fluorescent probes were loaded. After incubation at 37 °C for 20 min in the dark, the medium was replaced with fresh medium, and the cells were exposed to blue light irradiation for 2 min at a power density of 300 mW·cm^-2^ then immediately imaged using a fluorescence microscope (IX71, Olympus, Tokyo, Japan). For DHR123, λ_ex_ = 488 nm, the bandpass filter λ = 500-550 nm; For HPF, λ_ex_ = 488 nm, the bandpass filter λ = 500-550 nm.

### Mitochondrial membrane potential (∆ψ_m_) measurement

After adherent growth of A2058 cells and MHCC 97H cells cultured in 35-mm cell culture dishes, the cells were incubated with 5 μM T-T NPs for 4 h and exposed to blue light for 5 min. Then JC-10 Mitochondrial Membrane Potential Probe was used to detect potential changes in the ∆ψ_m_ in cells according to the instructions (HYCEZMBIO, HY222298). Cells were observed using fluorescence microscopy (IX71, Olympus, Tokyo, Japan). For JC-10 monomers, λ_ex_ = 490 nm, the bandpass filter λ = 505-555 nm; for JC-10 aggregates, λ_ex_ = 525 nm, the bandpass filter λ = 593-668 nm.

### Calcein-AM/T-T NPs dual fluorescence staining

Calcein-AM/PI staining is commonly used to differentiate live and dead cells, but PI overlaps with the fluorescence of the TTVPHA probe. Therefore, we used the fluorescence of T-T NPs as the self-monitoring fluorescence for whole cells. After A2058 cells and MHCC 97H cells in 35-mm cell culture dishes received different treatments, the cells were incubated with 2 µM calcein-AM for 20 min in the dark. Images were visualized and acquired using a fluorescence microscope (IX71, Olympus, Japan). For calcein-AM, λ_ex_ = 494 nm, the bandpass filter λ = 505-555 nm.

### Cell viability in the presence of different cell death inhibitors

After A2058 cells and MHCC 97H cells seeded in 96-well plates overnight, the cells were cultured with different cell death inhibitors (100 μM apoptosis inhibitor of Z-VAD-FMK, or 20 μM autophagy inhibitor of Chloroquine, or 50 μM ferroptosis inhibitor of Ferrostatin-1, or 50 μM necroptosis inhibitor of Necrostatin-1, or 5 μM pyroptosis inhibitor of 2-Bromohexadecanoic acid) for 3 h before incubated with 5 μM T-T NPs for 4 h and exposed to blue light for 5 min. After cultured for another 3 h in the dark, CCK8 was added and incubated for 2 h, and A450 was measured by Multimode Plate Reader (PerkinElmer Pte. Ltd., Singapore). All inhibitors were purchased from MedChemExpress.

### Lactate Dehydrogenase (LDH) assay

LDH release assay kit (Beyotime, C0016) was used to detect the extracellular LDH released in cell supernatants. A2058 cells and MHCC 97H cells were seeded in 35-mm culture dishes and cultured for 24 h. The resulting cells were treated under the following conditions: negative control group (without any treatment), only light group (exposed to blue laser for 5 min), only T-T NPs group (incubating with 5 μM T-T NPs for 4 h) and T-T NPs + Light (incubating with 5 μM T-T NPs for 4 h and exposed to blue laser for 5 min). After different treatments, the cell supernatants were collected for further measurement of LDH levels following the instructions, and A490 was measured by Multimode Plate Reader (PerkinElmer Pte. Ltd., Singapore).

### Adenosine Triphosphate (ATP) assay

ATP assay kit (Beyotime, S0027) was used to determine the intracellular ATP content. After A2058 cells and MHCC 97H cells treated based on the experimental protocol of LDH levels detection, the cells were collected for further detection of intracellular ATP according to the instructions, and bioluminescence was measured by Multimode Plate Reader (PerkinElmer Pte. Ltd., Singapore).

### Western blotting analysis

The western blot was completed according to a standard protocol. Total protein samples were extracted from A2058 cells and MHCC 97H cells using a mixture of RIPA and protease inhibitors. The sample protein concentration was measured by BCA protein assay kit and mixed with a 5× sample loading buffer boiling for 5 min at 95 °C. After SDS‒PAGE, the proteins were transferred to a 0.2 μm PVDF membrane. The membrane was blocked with 5% nonfat powdered milk solution for 1 h to allow for primary antibodies incubation overnight at 4 °C. After incubation with the secondary antibody for 1 h, the protein immunoreactivity was visualized by the chemiluminescence (ECL) substrate (Biosharp, BL523A) and imaged by ChemiDoc imaging system (Bio-Rad, Hercules, USA). The following antibodies were used: anti-cleaved caspase-3 (CST, 1:1000), anti-GSDME (Abcam, 1:900), anti-phospho-MLKL (ABclonal, 1:500), anti-BAX (Proteintech, 1:2000), anti-Bcl-2 (Proteintech, 1:5000), anti-Caspase 9 (Proteintech, 1:500), anti-PARP1 (Proteintech, 1:5000), anti-RIPK1 (Proteintech, 1:1000), anti-phospho-RIPK1 (Proteintech, 1:2000), anti-MLKL (Proteintech, 1:5000), anti-GPX4 (Proteintech, 1:1000) and anti-GAPDH (Proteintech, 1:5000).

Tumor tissues (250 mg) were homogenized on ice in 1 mL of RIPA supplemented with protease and phosphatase inhibitors using a tissue homogenizer (Physcotron, NS-50; Microtec, Chiba, Japan) then centrifuged for 30 min at 12,000×g and 4 °C to isolate the whole protein. The subsequent experimental steps were the same as described above.

### Intracellular GSH detection

GSH assay kit (Beyotime, S0053) was used to assess the intracellular GSH level. A2058 cells and MHCC 97H cells were seeded in 35-mm culture dishes and cultured for 24 h. After A2058 cells and MHCC 97H cells treated based on the experimental protocol of LDH levels detection, the cells were collected for further measurement of GSH content according to the instructions, and A412 was measured by Multimode Plate Reader (PerkinElmer Pte. Ltd., Singapore).

### Malondialdehyde (MDA) level detection

The MDA levels were detected by an MDA assay kit (Beyotime, S0131S). After A2058 cells and MHCC 97H cells treated based on the experimental protocol of GSH levels detection, the cells were collected and centrifugation. The protein concentrations were measured by a BCA protein assay kit, and the MDA concentrations were detected according to the instruction. The MDA level was calculated by the ratio of MDA concentrations and protein concentrations.

### Measurement of lipid peroxidation

C11-BODIPY (581/591) dye (Thermo Fisher, D3861) was used to evaluate the intracellular lipid peroxidation content. Cells from each experimental group were incubated with PBS containing C11-BODIPY probe for 60 min in the dark. The fluorescence signal images were obtained by CLSM (Nikon Corporation, Japan). For C11-BODIPY, λ_ex_ = 488 nm, the bandpass filter λ = 510 nm.

### Intracellular Fe^2+^ detection

Cell Ferrous Iron Colorimetric Assay Kit (Elabscience, E-BC-K881-M) was used to detect the intracellular Fe^2+^ level. The resulting samples were collected from different treatment groups and further experimented following the instructions. The A593 was measured by Multimode Plate Reader (PerkinElmer Pte. Ltd., Singapore).

### Transmission Electron Microscope (TEM) analysis

The morphology of mitochondria in ferroptosis cells was observed using TEM according to standard procedure. The treated A2058 cells and MHCC 97H cells were harvested and fixed in 2.5% glutaraldehyde buffer (Biosharp, BL911A) overnight. The cells were dried using a CO_2_ critical point dryer (Leica EM CPD300, Leica Microsystems, Wetzlar, Germany), stained with 2% uranium acetate saturated alcohol solution and 2.6% Lead citrate at room temperature, rinsed and observed by TEM (HITACHI, HT7700).

### Animal tumor model

4-week-old male BALB/c nude mice were purchased from SiPeiFu (SPF) Biotechnology Co., Ltd. (Beijing, China) and housed under specific pathogen-free (SPF) conditions at Tongji Medical College Animal Experimentation, and the experiments were performed in compliance with the guidelines established by the Ethics Committee of Tongji Medical College, HUST, Wuhan, China ([2024] IACUC Number: 4080). The tumor model was established by injecting 1 × 10^7^ A2058 cells into the right axilla of mouse. Tumor growth and body weights were measured every two days, and tumor volume was calculated using the formula: volume = ((tumor length) × (tumor width)^2^)/2.

### Hemolysis assay

One mL blood samples were obtained from BALB/c mice and diluted with 2 mL PBS. Red blood cells (RBCs) were separated from the serum by centrifugation (2000 rpm, 10 min). After washing three times with PBS, the RBCs were then diluted with 10 mL of PBS. A suspension of RBCs (30 μL) was mixed with 120 μL of saline (negative control), distilled water (positive control), and T-T NPs at different concentrations (5, 10, and 20 μg/mL). After incubating at 37 °C for 1 h, 2 h and 3 h, the mixtures were centrifuged at 12000 rpm for 10 min. The hemolysis images were taken and then the supernatants (100 μL) of each sample were added to a 96-well plate, and A570 was measured by Multimode Plate Reader (PerkinElmer Pte. Ltd., Singapore).

### *In vivo* biosafety analysis

Healthy male Balb/c mice were divided into 2 groups (n=3). One group of mice was intravenously injected with 125 μL T-T NPs (4 mg/mL) and the other group treated with PBS was used as control. After 7 days, the blood samples of mice were collected for blood chemistry tests, and the main organs of the mice (heart, liver, spleen, lung, and kidney) were acquired and stained using H&E for histological observations.

### *In vivo* fluorescence imaging and biodistribution analysis

The tumor-bearing mice were established as described above. Real-time fluorescence imaging was performed using an In-Vivo FX PRO (BRUKER, Germany) at 8, 24 and 48 h after injected 125 μL of T-T NPs (4 mg/mL) via the tail vein. Then the major organs (liver, kidney, spleen, heart, and lung) and tumor were collected at 24 h post-injection and their fluorescence intensity were measured by an *In*-*Vivo* FX PRO (BRUKER, Germany).

### *In vivo* anti-tumor efficacy of T-T NPs

The tumor-bearing mice were established as described above. When the tumor size reached 60 mm^3^, A2058 tumor-bearing mice were divided into 4 groups (n=5): (1) PBS Group: injected with 125 μL of PBS; (2) Light Group: laser only (490 nm, 960 mW·cm^-2^, 15 min); (3) T-T NPs Group: only injected with 125 μL of T-T NPs (4 mg/mL); (4) T-T NPs plus light Group: injected with 125 μL of T-T NPs (4 mg/mL) and exposed to irradiation (490 nm, 960 mW·cm^-2^, 15 min). The T-T NPs were administered on days 0 and 3, and laser irradiation was performed 24 h after injection. Tumor sizes were measured for 14 days after treatment and the mice were sacrificed.

### Immunohistochemical fluorescence staining

Tumor tissues were acquired and reserved in 4% paraformaldehyde (Biosharp, BL539A), embedded in paraffin and cut into 3 μm sections, followed by deparaffinized and rehydrated. Antigen retrieval was achieved at high temperatures in a pressure cooker using citrate buffer (pH=6.0). Primary antibodies against Ki67 (Proteintech, 1:400) were used for immunohistochemical staining. The paraffin-embedded sections of the tumors were stained with hematoxylin and eosin (H&E) and subjected to TUNEL. Images were obtained by scanning with a fluorescence microscope (IX71, Olympus, Tokyo, Japan).

### Statistical analysis

GraphPad Prism 9 software was used to analyse data performed in the form of mean ± standard deviation (SD) or standard error of the mean (SEM). One-way analysis of variance ANOVA with Tukey's test was used for multiple comparisons when more than two groups were compared, and two-tailed Student's t tests were used for two-group comparisons and significance was considered at a p-value < 0.05. The notation of an asterisk (*) indicates statistical significance observed between the respective bars (ns, no statistical difference; *, p < 0.05; **, p < 0.01; ***, p < 0.001; ****, p < 0.0001).

## Supplementary Material

Supplementary materials and methods, figures.
