# Supplementary material for: Oxidative stress-mediated PANoptosis and ferroptosis: Exploration of multimodal cell death triggered by an AIE-active nano-photosensitizer via photodynamic therapy
Source: Theranostics. 2025 Jun 9;15(14):6665–85. doi: 10.7150/thno.111635 (PMC12203667; doi:10.7150/thno.111635)
Supplement: Supplementary file 1 — Supplementary materials and methods, figures. [file thnov15p6665s1.pdf]

Supporting Information

1. Materials and Methods

1.1. Materials and Instruments

TTVPHA was selected from ASBase as an AIE-characteristic PS (https://www.asbase.cn/Search/info.html?id=1901), and synthesized according to our previous report [1]. DSPE-PEG$_{2000}$-TAT and DSPE-PEG$_{2000}$ were purchased from Xi'an Ruixi Biological Technology Co., Ltd (Xian, China). The human melanoma cell line A2058 was obtained from the Cell Bank of the Chinese Academy of Sciences (Shanghai, China) and the human hepatocellular carcinoma cell line MHCC 97H was obtained from Cellverse (iCell) Bioscience Technology Co., Ltd (Shanghai, China). Fetal bovine serum (FBS) and Dulbecco's modified Eagle's medium (DMEM) were purchased from Gibco (Grand Island, USA). Cell Counting Kit-8 (CCK8), 2',7'-Dichlorofluorescin diacetate (DCFH-DA) and JC-10 were obtained from HYCEZMBIO (Wuhan, China). Dihydrorhodamine 123 (DHR 123) was purchased from GlpBio Technology (GC30581, USA). Hydroxyphenyl Fluorescein (HPF) was obtained from MedChemExpress (MCE) (HY-111330, Shanghai, China). 9.10-Anthracenediyl-bis(methylene)dimalonic Acid (ABDA) was obtained from Maokang Technology (MK4822-50MG, Shanghai, China). C11-BODIPY (581/591) dye and PVDF transfer membranes were purchased from Thermo Fisher (Waltham, MA, USA). MitoTracker Green (MTG), Lyso-Tracker Green (LTG), DAPI, LDH release assay kit, enhanced ATP assay kit, Calcein-AM/PI dual staining kit, GSH assay kit, MDA assay kit, BCA protein assay kit, general protease inhibitor cocktail, general phosphatase inhibitor cocktail and nonfat powdered milk were purchased from Beyotime (Jiangsu, China). ECL substrate, RIPA lysis buffer, 4% paraformaldehyde, 2.5% glutaraldehyde buffer and DMSO were purchased from Biosharp (Hefei, China). GAPDH, BAX, Bcl-2, Caspase 9, PARP1, RIPK1, phospho-RIPK1, MLKL, GPX4, and HRP-labeled anti-rabbit/mouse secondary antibodies were purchased from Proteintech (Wuhan, China). Cleaved caspase-3 was purchased from Cell Signaling Technology (Danvers, MA, USA). GSDME was purchased from Abcam (Cambridge, MA, USA). Phospho-MLKL was purchased from ABclonal Technology (Wuhan, China). RIPA buffer, TBS and PBS were purchased from Servicebio Technology Co., Ltd. (Wuhan, China). The 5×sample loading buffer, protein ladder and 12.5% gel kit were purchased from Epizyme (Shanghai, China). Cell Ferrous Iron Colorimetric Assay Kit was

purchased from Elabscience Biotechnology Co., Ltd. (Wuhan, China). All biological inhibitors were obtained from MedChemExpress (MCE) (Shanghai, China). UV–vis absorption spectra was measured using a Shimadzu UV-2600 spectrophotometer and Photoluminescence (PL) spectra was recorded using a Horiba Fluoromax-4 spectrofluorometer.

1.2. Preparation and Characteristics of T-T NPs

1-(5-Carboxypentyl)-4-methylpyridin-1-ium bromide (72 mg, 0.25 mmol), 5-(4-(diphenylamino)phenyl)thiophene-2-carbaldehyde (80 mg, 0.25 mmol) and piperidine (200 μL) were mixed with ethanol (10 mL). The reaction mixture was heated and refluxed at 79 °C for 2 h. After cooling to room temperature, the solvent was removed under reduced pressure. The crude product was purified using silica-gel chromatography withCH_2Cl_2/CH_3OH= 80/20 as the eluent to afford TTVPHA as a red powder with a yield of 55.8% (87 mg). ^{1}H NMR (400 MHz, MeOD) δ 8.69 (d, J = 6.9 Hz, 2H), 8.05 (dd, J = 11.4, 8.1 Hz, 3H), 7.59-7.54 (m, 2H), 7.44 (d, J = 3.9 Hz, 1H), 7.37-7.27 (m, 5H), 7.13-6.98 (m, 9H), 4.47 (t, J = 7.4 Hz, 2H), 2.18 (t, J = 7.1 Hz, 2H), 2.04-1.95 (m, 2H), 1.67 (dt, J = 14.7, 7.2 Hz, 2H), 1.41 (dd, J = 15.1, 7.9 Hz, 2H) (**Figure S1**)

The TTVPHA (1 mg) and DSPE-PEG_{2000}-TAT (3 mg) with DSPE-PEG_{2000} (mass ratio = 1:1) were dissolved together in 1 mL methanol. It was slowly dripped into 9 mL dd water with a long needle in an ice bath and was sonicated (20% output power for 10 min, SCIENTZ-II D ultrasonicator) to prepare T-T NPs solution. After filtration through a 0.45 μm membrane, the solution was further concentrated by ultrafiltration centrifugation at 4 °C (5000 rpm, 30 min). The concentration of TTVPHA in the NPs was measured by comparing the absorption at 478 nm of the NPs diluted in methanol to a known concentration of TTVPHA, according to the Lambert-Beer law. Then, it was diluted with PBS to a certain concentration for subsequent experiments.

Photoluminescence (PL) spectra and UV-visible absorption spectroscopy were used to evaluate an aqueous solution of T-T NPs. The T-T NPs' dimensions and surface potential were measured by dynamic light scattering (DLS), and their nanoscopic morphology was confirmed through transmission electron microscopy (TEM). To assess the stability of the nanoparticles, the size distribution of T-T NPs was examined after varying storage times.

1.3. Total ROS Generation Efficiency of T-T NPs

2',7'-dichlorodihydrofluorescein diacetate (DCFH-DA) was first dissolved in ethanol to prepare a 1 mM stock solution. Subsequently, 500 µL of this stock was mixed with 2 mL of 0.01 M NaOH and allowed to react for 30 min at room temperature in the dark. The reaction was then neutralized by adding 10 mL of PBS, resulting in a 40 µM DCFH solution, which was stored at -20 °C in the dark. For the assay, DCFH (10 µM) was combined with T-T NPs (20 µM) in PBS, and after exposure to white light, the fluorescence intensity at 525 nm, upon excitation at 488 nm, was measured to quantify ROS production.

1.4. Type I and Type II ROS Generation Efficiency of T-T NPs

Dihydrorhodamine 123 (DHR123) was used as an $O_2^{\bullet-}$ indicator. A solution of DHR123 (10 µM) and T-T NPs (20 µM) in DMSO/PBS (1:99) was prepared, and after exposure to white light, the fluorescence increase at 528 nm upon excitation at 485 nm was measured.

For •OH detection, Hydroxyphenyl Flavin (HPF) was employed. HPF (10 µM) and T-T NPs (20 µM) were mixed in DMF/PBS (1:99), and following white light irradiation, the fluorescence enhancement at 525 nm with excitation at 490 nm was assessed.

For $^1O_2$ detection, 9.10-Anthracenediyl-bis(methylene)dimalonic Acid (ABDA) was used. ABDA (50 µM) was mixed with T-T NPs (20 µM) in PBS containing 1% DMSO. After exposure to white light, the changes in absorbance between 350-410 nm were measured.

1.5. Cell Culture

A2058 cells and MHCC 97H cells were cultured in high-glucose Dulbecco's modified Eagle's medium (DMEM, Gibco) with 10% fetal bovine serum (FBS, Gibco) and 1% penicillin-streptomycin (P/S). The cells were all cultured in a constant temperature incubator at 37 °C with 5% CO_2 and 90% relative humidity.

1.6. Cell Uptake and Fluorescence Imaging

For the detection of the intracellular distribution of T-T NPs after various incubation times, the cells were inoculated in laser confocal dishes and cultured overnight for adhesion. The culture medium was removed, and the cells were incubated with 5 µM T-T NPs for 10 min, 30 min, 1 h, 2 h and 4 h, respectively. After washing three times with PBS, the cells were observed and imaged directly using a confocal laser scanning microscope (CLSM, Nikon Corporation, Japan). For T-T NPs, λ_{ex} = 488 nm, the bandpass filter λ = 552-617 nm.

To investigate the intracellular distribution of NPs with different incubation concentrations, the cells were inoculated in laser confocal dishes overnight then incubated with 2.5, 5 and 10 μM T-T NPs for 4 h, respectviely. The blocking group was pretreated with genistein (MCE, HY-14596) for 2 h before incubating T-T NPs. After rising with PBS three times, the cells were fixed with 4% paraformaldehyde (Biosharp, BL539A) for 10 min and were stained with 10 μg/mL of Actin-Tracker Green (F-actin, Beyotime, C2201S) at 37 °C in the dark for 40 min. Immediately following three washes with PBS, the cell nucleus was labeled with 5 μg/mL DAPI (Invitrogen, 62248) for 5 min. After washing three times with PBS, the cells were subjected to imaging analysis directly using CLSM (Nikon Corporation, Japan). For DAPI, λ_{ex} = 405 nm, the bandpass filter λ = 425-475 nm. For F-actin, λ_{ex} = 488 nm, the bandpass filter λ = 500-530 nm. Imaging analysis was performed directly using NIS-Eliments software (Nikon, Japan).

1.7. Quantitative Analysis of T-T NPs Uptake Using Flow Cytometry

The A2058 cells and MHCC 97H cells were homogeneously inoculated in cell culture dishes and placed in a cell culture incubator overnight for adherent growth. After washing once with PBS, each dish was added with serum-free medium containing different concentrations of T-T NPs (2.5, 5 and 10 μM) incubating for 4 h. After washing three times with PBS, the cells in culture dishes were digested into flow tubes using trypsin and resuspended in 200 μL of PBS. Flow cytometry (BD Falcon, San Jose, CA, USA) was immediately used to quantitate the intracellular fluorescence intensity, and the data were analyzed using FlowJo 10.8.1. For T-T NPs, λ_{ex} = 488 nm， the bandpass filter λ = 605 nm.

1.8. Colocalization Analysis between T-T NPs and Mitochondrion Tracker and Lysosome Tracker

The A2058 cells and MHCC 97H cells were homogeneously seeded in 35-mm confocal dishes, cultured overnight, and incubated with 5 μM T-T NPs for 1 h or 4 h. After three washes with PBS, each dish was incubated with Mito-Tracker Green (MTG, Beyotime, C1048) or Lyso-Tracker Green (LTG, Beyotime, C1047S) for 45 min. Real-time imaging was captured using CLSM (Nikon Corporation, Japan), and the co-localization data were analyzed using ImageJ (National Institutes of Health Free Software, USA). For MTG, λ_{ex} = 488 nm, the bandpass filter λ = 500-530 nm. For LTG, λ_{ex} = 488 nm, the bandpass filter λ = 500-530 nm.

1.9. Cell Viability Assay

Cell Viability of A2058 cells and MHCC 97H cells after different treatments was detected by CCK8 assay. To assess the dark toxicity of T-T NPs, after culturing 1×10^4 cells overnight 96-well plates, the medium containing a series of concentrations of T-T NPs (0, 2.5, 5, 7.5, or 10 μM) for 4 h under hypoxic or normoxic conditions, and after washing three times with PBS, the plates were continuously cultured for 24 h. Subsequently, the original medium was removed and the medium containing CCK8 was added and incubated for 2 h, and A450 was measured by Multimode Plate Reader (PerkinElmer Pte. Ltd., Singapore).

To assess the cytotoxicity induced by PDT under different light irradiation times, the cells in the 96-well plate were incubated with 5 μM T-T NPs for 4 h under hypoxic or normoxic conditions in the dark then exposed to blue laser irradiation (300 mW·cm ^{2}) for different times (0, 2, 5, 8, and 10 min). The cells were cultured for 24 h, and the cell viability was measured according to the above description.

When CCK8 was used to detect the photodynamic effects in different treatment groups, the resulting cells were treated under the following conditions: negtive control group (without any treatment), only light group (exposed to blue laser for 5 min), only T-T NPs group (incubating with 5 μM T-T NPs for 4 h) and T-T NPs + Light (incubating with 5 μM T-T NPs for 4 h and exposed to blue laser for 5 min). The cells were suequentially cultured for 24 h, and the cell viability was measured according to the above description.

1.10. Intracellular ROS Detection

The total ROS generation ability induced by PDT was detected by the fluorescent probe DCFH-DA according to the manufacturer's instructions for the ROS detection kit (HYCEZMBIO, 040-100T). The DHR123 and HPF were used as $O_2^{\bullet-}$ and •OH indicators, respectively.

For normoxic conditions, after attachment of A2058 cells and MHCC 97H cells cultured in 35-mm cell culture plates, the cells were incubated with 5 μM T-T NPs for 4 h in a 21% O_2 atmosphere. Then the DCFH-DA fluorescent probe was loaded. After incubation at 37 °C for 20 min in the dark, the medium was replaced with fresh medium, and the cells were exposed to blue light irradiation for 2 min at a power density of 300 mW·cm ^{2} then immediately imaged using a fluorescence microscope (IX71, Olympus, Tokyo, Japan). The cells incubated with Rosup solution

for 30 min were treated as the positive control. For DCFH-DA, λ_{ex} = 488 nm, the bandpass filter λ = 500-550 nm.

For hypoxic condition, after attachment of A2058 cells and MHCC 97H cells cultured in 35-mm cell culture plates, the cells were incubated with 5 μM T-T NPs for 4 h in a 1% O_2 atmosphere. Then the DHR123 and HPF fluorescent probes were loaded. After incubation at 37 °C for 20 min in the dark, the medium was replaced with fresh medium, and the cells were exposed to blue light irradiation for 2 min at a power density of 300 mW·cm ^{2} then immediately imaged using a fluorescence microscope (IX71, Olympus, Tokyo, Japan). For DHR123, λ_{ex} = 488 nm, the bandpass filter λ = 500-550 nm; For HPF, λ_{ex} = 488 nm, the bandpass filter λ = 500-550 nm.

1.11. Mitochondrial Membrane Potential ($\Delta\psi_m$) Measurement

After adherent growth of A2058 cells and MHCC 97H cells cultured in 35-mm cell culture dishes, the cells were incubated with 5 μM T-T NPs for 4 h and exposed to blue light for 5 min. Then JC-10 Mitochondrial Membrane Potential Probe was used to detect potential changes in the $\Delta\psi_m$ in cells according to the instructions (HYCEZMBIO, HY222298). Cells were observed using fluorescence microscopy (IX71, Olympus, Tokyo, Japan). For JC-10 monomers, λ_{ex} = 490 nm, the bandpass filter λ = 505-555 nm; for JC-10 aggregates, λ_{ex} = 525 nm, the bandpass filter λ = 593-668 nm.

1.12. Calcein-AM/T-T NPs Dual Fluorescence Staining

Calcein-AM/PI staining is commonly used to differentiate live and dead cells, but PI overlaps with the fluorescence of the TTVPHA probe. Therefore, we used the fluorescence of T-T NPs as the self-monitoring fluorescence for whole cells. After A2058 cells and MHCC 97H cells in 35-mm cell culture dishes received different treatments, the cells were incubated with 2 μM calcein-AM for 20 min in the dark. Images were visualized and acquired using a fluorescence microscope (IX71, Olympus, Japan). For calcein-AM, λ_{ex} = 494 nm, the bandpass filter λ = 505-555 nm.

1.13. Cell Viability in the Presence of Different Cell Death Inhibitors

After A2058 cells and MHCC 97H cells seeded in 96-well plates overnight, the cells were cultured with different cell death inhibitors (100 μM apoptosis inhibitor of Z-VAD-FMK, or 20 μM autophagy inhibitor of Chloroquine, or 50 μM ferroptosis inhibitor of Ferrostatin-1, or 50 μM necroptosis inhibitor of Necrostatin-1, or 5 μM pyroptosis inhibitor of 2-Bromohexadecanoic acid)

for 3 h before incubated with 5 μM T-T NPs for 4 h and exposed to blue light for 5 min. After cultured for another 3 h in the dark, CCK8 was added and incubated for 2 h, and A450 was measured by Multimode Plate Reader (PerkinElmer Pte. Ltd., Singapore). All inhibitors were purchased from MedChemExpress.

1.14. Lactate Dehydrogenase (LDH) Assay

LDH release assay kit (Beyotime, C0016) was used to detect the extracellular LDH released in cell supernatants. A2058 cells and MHCC 97H cells were seeded in 35-mm culture dishes and cultured for 24 h. The resulting cells were treated under the following conditions: negative control group (without any treatment), only light group (exposed to blue laser for 5 min), only T-T NPs group (incubating with 5 μM T-T NPs for 4 h) and T-T NPs + Light (incubating with 5 μM T-T NPs for 4 h and exposed to blue laser for 5 min). After different treatments, the cell supernatants were collected for further measurement of LDH levels following the instructions, and A490 was measured by Multimode Plate Reader (PerkinElmer Pte. Ltd., Singapore).

1.15. Adenosine Triphosphate (ATP) Assay

ATP assay kit (Beyotime, S0027) was used to determine the intracellular ATP content. After A2058 cells and MHCC 97H cells treated based on the experimental protocol of LDH levels detection, the cells were collected for further detection of intracellular ATP according to the instructions, and bioluminescence was measured by Multimode Plate Reader (PerkinElmer Pte. Ltd., Singapore).

1.16. Western blotting Analysis

The western blot was completed according to a standard protocol. Total protein samples were extracted from A2058 cells and MHCC 97H cells using a mixture of RIPA and protease inhibitors. The sample protein concentration was measured by BCA protein assay kit and mixed with a 5× sample loading buffer boiling for 5 min at 95 °C. After SDS–PAGE, the proteins were transferred to a 0.2 μm PVDF membrane. The membrane was blocked with 5% nonfat powdered milk solution for 1 h to allow for primary antibodies incubation overnight at 4 °C. After incubation with the secondary antibody for 1 h, the protein immunoreactivity was visualized by the chemiluminescence (ECL) substrate (Biosharp, BL523A) and imaged by ChemiDoc imaging system (Bio-Rad, Hercules, USA). The following antibodies were used: anti-cleaved caspase-3 (CST, 1:1000), anti-GSDME (Abcam,

1:900), anti-phospho-MLKL (ABclonal, 1:500), anti-BAX (Proteintech, 1:2000), anti-Bcl-2 (Proteintech, 1:5000), anti-Caspase 9 (Proteintech, 1:500), anti-PARP1 (Proteintech, 1:5000), anti-RIPK1 (Proteintech, 1:1000), anti-phospho-RIPK1 (Proteintech, 1:2000), anti-MLKL (Proteintech, 1:5000), anti-GPX4 (Proteintech, 1:1000) and anti-GAPDH (Proteintech, 1:5000).

Tumor tissues (250 mg) were homogenized on ice in 1 mL of RIPA supplemented with protease and phosphatase inhibitors using a tissue homogenizer (Physcotron, NS-50; Microtec, Chiba, Japan) then centrifuged for 30 min at 12,000×g and 4 °C to isolate the whole protein. The subsequent experimental steps were the same as described above.

1.17. Intracellular GSH Detection

GSH assay kit (Beyotime, S0053) was used to assess the intracellular GSH level. A2058 cells and MHCC 97H cells were seeded in 35-mm culture dishes and cultured for 24 h. After A2058 cells and MHCC 97H cells treated based on the experimental protocol of LDH levels detection, the cells were collected for further measurement of GSH content according to the instructions, and A412 was measured by Multimode Plate Reader (PerkinElmer Pte. Ltd., Singapore).

1.18. Malondialdehyde (MDA) Level Detection

The MDA levels were detected by an MDA assay kit (Beyotime, S0131S). After A2058 cells and MHCC 97H cells treated based on the experimental protocol of GSH levels detection, the cells were collected and centrifugation. The protein concentrations were measured by a BCA protein assay kit, and the MDA concentrations were detected according to the instruction. The MDA level was calculated by the ratio of MDA concentrations and protein concentrations.

1.19. Measurement of Lipid Peroxidation

C11-BODIPY (581/591) dye (Thermo Fisher, D3861) was used to evaluate the intracellular lipid peroxidation content. Cells from each experimental group were incubated with PBS containing C11-BODIPY probe for 60 min in the dark. The fluorescence signal images were obtained by CLSM (Nikon Corporation, Japan). For C11-BODIPY, λ_{ex} = 488 nm, the bandpass filter λ = 510 nm.

1.20. Intracellular Fe^{2+} Detection

Cell Ferrous Iron Colorimetric Assay Kit (Elabscience, E-BC-K881-M) was used to detect the intracellular Fe^{2+} level. The resulting samples were collected from different treatment groups and

further experimented following the instructions. The A593 was measured by Multimode Plate Reader (PerkinElmer Pte. Ltd., Singapore).

1.21. Transmission Electron Microscope (TEM) Analysis

The morphology of mitochondria in ferroptosis cells was observed using TEM according to standard procedure. The treated A2058 cells and MHCC 97H cells were harvested and fixed in 2.5% glutaraldehyde buffer (Biosharp, BL911A) overnight. The cells were dried using a CO_2 critical point dryer (Leica EM CPD300, Leica Microsystems, Wetzlar, Germany), stained with 2% uranium acetate saturated alcohol solution and 2.6% Lead citrate at room temperature, rinsed and observed by TEM (HITACHI, HT7700).

1.22. Animal Tumor Model

4-week-old male BALB/c nude mice were purchased from SiPeiFu (SPF) Biotechnology Co., Ltd. (Beijing, China) and housed under specific pathogen-free (SPF) conditions at Tongji Medical College Animal Experimentation, and the experiments were performed in compliance with the guidelines established by the Ethics Committee of Tongji Medical College, HUST, Wuhan, China ([2024] IACUC Number: 4080). The tumor model was established by injecting 1×10^7 A2058 cells into the right axilla of mouse. Tumor growth and body weights were measured every two days, and tumor volume was calculated using the formula: volume = ((tumor length) × (tumor width)^2)/2.

1.23. Hemolysis Assay

One mL blood samples were obtained from BALB/c mice and diluted with 2 mL PBS. Red blood cells (RBCs) were separated from the serum by centrifugation (2000 rpm, 10 min). After washing three times with PBS, the RBCs were then diluted with 10 mL of PBS. A suspension of RBCs (30 μL) was mixed with 120 μL of saline (negative control), distilled water (positive control), and T-T NPs at different concentrations (5, 10, and 20 μg/mL). After incubating at 37 °C for 1 h, 2 h and 3 h, the mixtures were centrifuged at 12000 rpm for 10 min. The hemolysis images were taken and then the supernatants (100 μL) of each sample were added to a 96-well plate, and A570 was measured by Multimode Plate Reader (PerkinElmer Pte. Ltd., Singapore).

1.24. *In vivo* Biosafety Analysis

Healthy male Balb/c mice were divided into 2 groups (n=3). One group of mice was

intravenously injected with 125 μL T-T NPs (4 mg/mL) and the other group treated with PBS was used as control. After 7 days, the blood samples of mice were collected for blood chemistry tests, and the main organs of the mice (heart, liver, spleen, lung, and kidney) were acquired and stained using H&E for histological observations.

1.25. *In Vivo* Fluorescence Imaging and Biodistribution Analysis

The tumor-bearing mice were established as described above. Real-time fluorescence imaging was performed using an In-Vivo FX PRO (BRUKER, Germany) at 8, 24 and 48 h after injected 125 μL of T-T NPs (4 mg/mL) *via* the tail vein. Then the major organs (liver, kidney, spleen, heart, and lung) and tumor were collected at 24 h post-injection and their fluorescence intensity were measured by an In-Vivo FX PRO (BRUKER, Germany).

1.26. *In Vivo* Anti-tumor Efficacy of T-T NPs

The tumor-bearing mice were established as described above. When the tumor size reached 60 mm^3, A2058 tumor-bearing mice were divided into 4 groups (n=5): (1) PBS Group: injected with 125 μL of PBS; (2) Light Group: laser only (490 nm, 960 $mW{\cdot}cm^{-2}$, 15 min); (3) T-T NPs Group: only injected with 125 μL of T-T NPs (4 mg/mL); (4) T-T NPs plus light Group: injected with 125 μL of T-T NPs (4 mg/mL) and exposed to irradiation (490 nm, 960 $mW{\cdot}cm^{-2}$, 15 min). The T-T NPs were administrated on days 0 and 3, and laser irradiation was performed 24 h after injection. Tumor sizes were measured for 14 days after treatment and the mice were sacrificed.

1.27. Immunohistochemical Fluorescence Staining

Tumor tissues were acquired and reserved in 4% paraformaldehyde (Biosharp, BL539A), embedded in paraffin and cut into 3 μm sections, followed by deparaffinized and rehydrated. Antigen retrieval was achieved at high temperatures in a pressure cooker using citrate buffer (pH=6.0). Primary antibodies against Ki67 (Proteintech, 1:400) were used for immunohistochemical staining. The paraffin-embedded sections of the tumors were stained with hematoxylin and eosin (H&E) and subjected to TUNEL. Images were obtained by scanning with a fluorescence microscope (IX71, Olympus, Tokyo, Japan).

1.28. Statistical Analysis

GraphPad Prism 9 software was used to analyse data performed in the form of mean ± standard

deviation (SD) or standard error of the mean (SEM). One-way analysis of variance ANOVA with Tukey's test was used for multiple comparisons when more than two groups were compared, and two-tailed Student's *t* tests were used for two-group comparisons and significance was considered at a p-value < 0.05. The notation of an asterisk (*) indicates statistical significance observed between the respective bars (ns, no statistical difference; *, $p < 0.05$; **, $p < 0.01$; ***, $p < 0.001$; ****, $p < 0.0001$).

2. Supplementary Figures

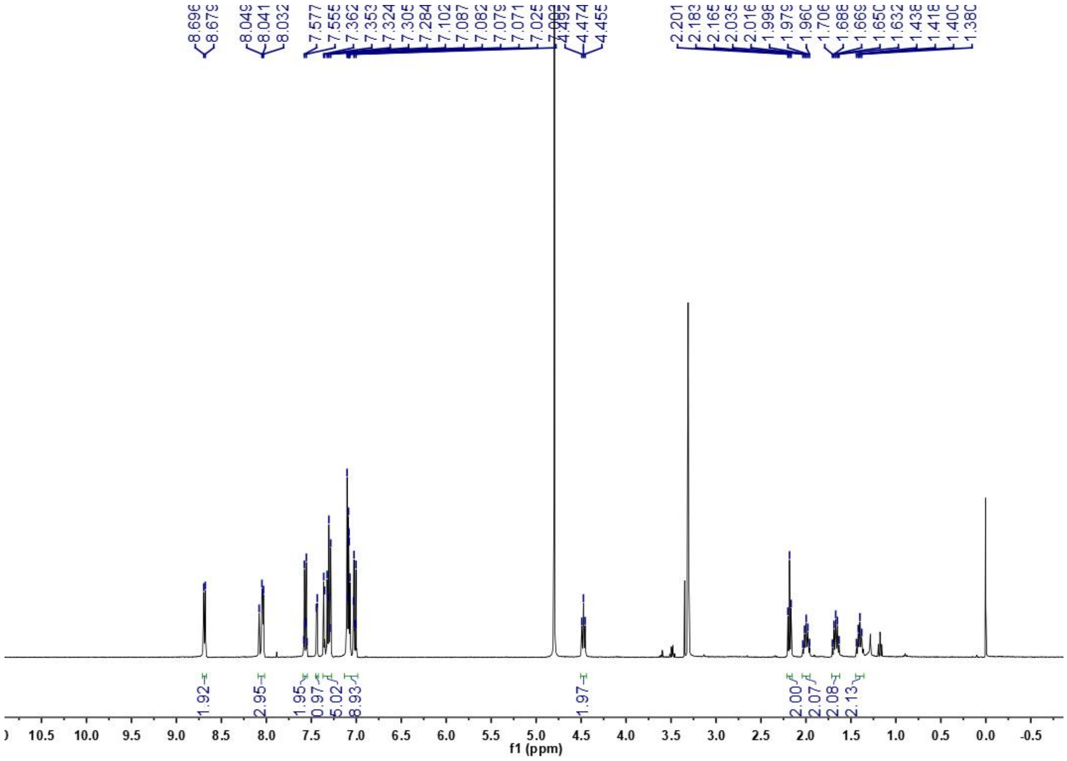

Figure S1. The ^{1}H NMR spectra of compound TTVPHA in MeOD.

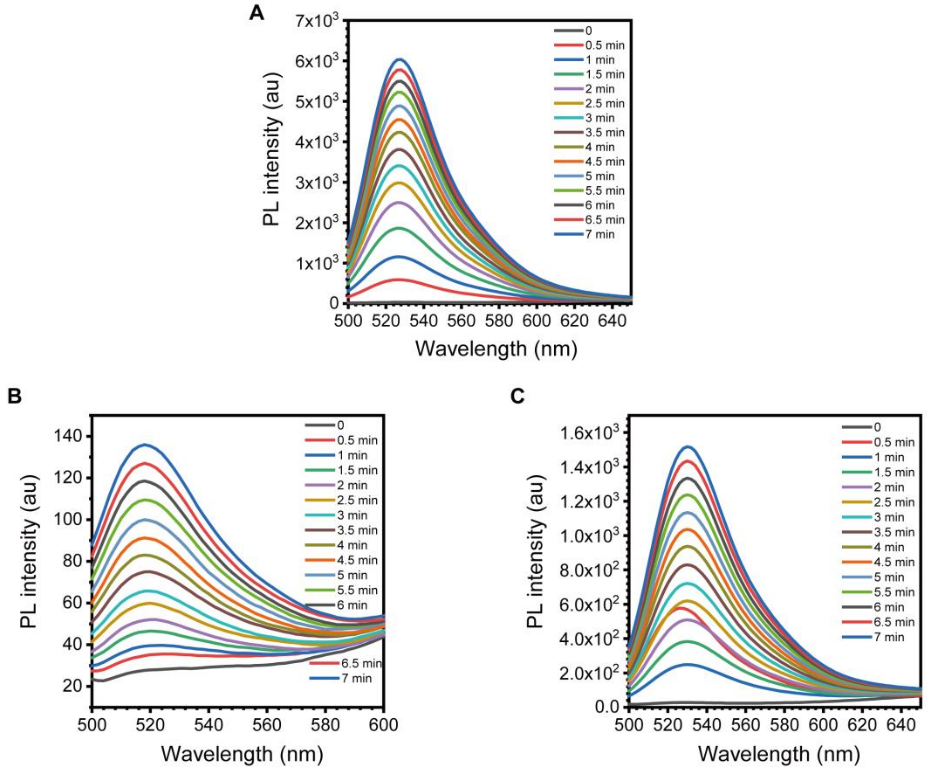

Figure S2. (A) Thefluorescence emission spectra of DcFH (for detection of total Ros) after incubation with T-T NPs under white light irradiation (20 mW·cm^{-2}). **(B)** HPF probe sensing •OH generation in aqueous solution for

T-T NPs with white light irradiation (20 mW·cm^{-2}). **(C)** DHR123 probe sensing $O_2^{\bullet-}$ generation in aqueous solution for T-T NPs with white light irradiation (20 mW·cm^{-2}).

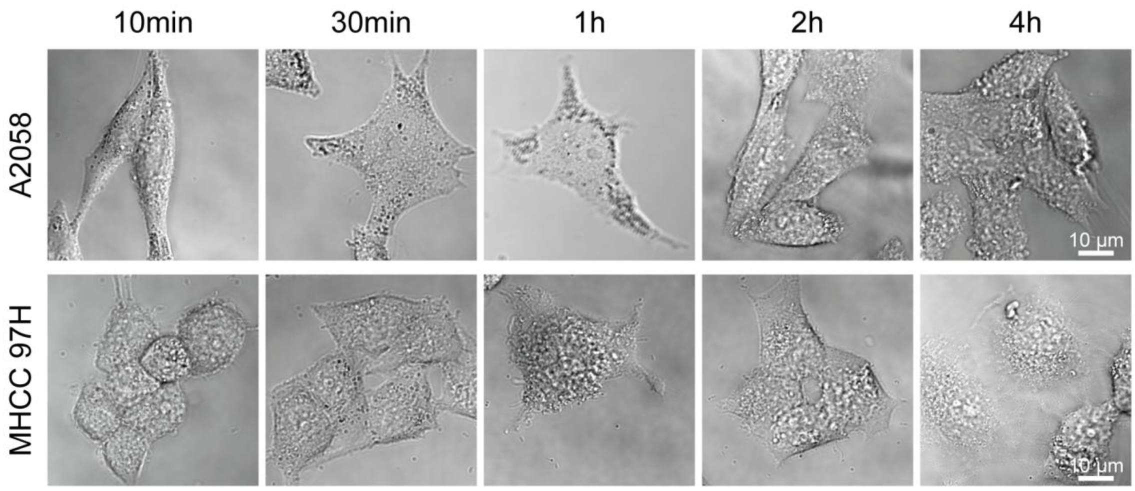

Figure S3. Bright images of A2058 and MHCC 97H cells with different incubation time monitored by confocal laser scanning microscopy (CLSM).

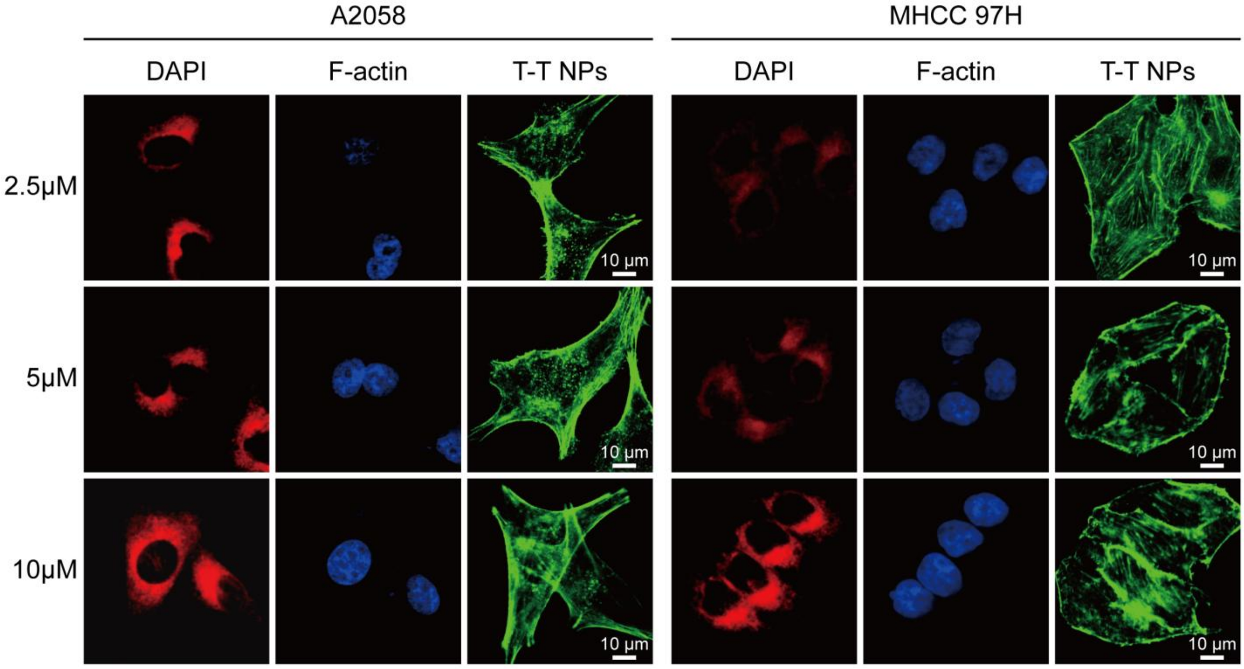

Figure S4. Fluorescence imaging of intracellular T-T NPs with a series of concentrations in A2058 and MHCC 97H cells for 4 h under CLSM.

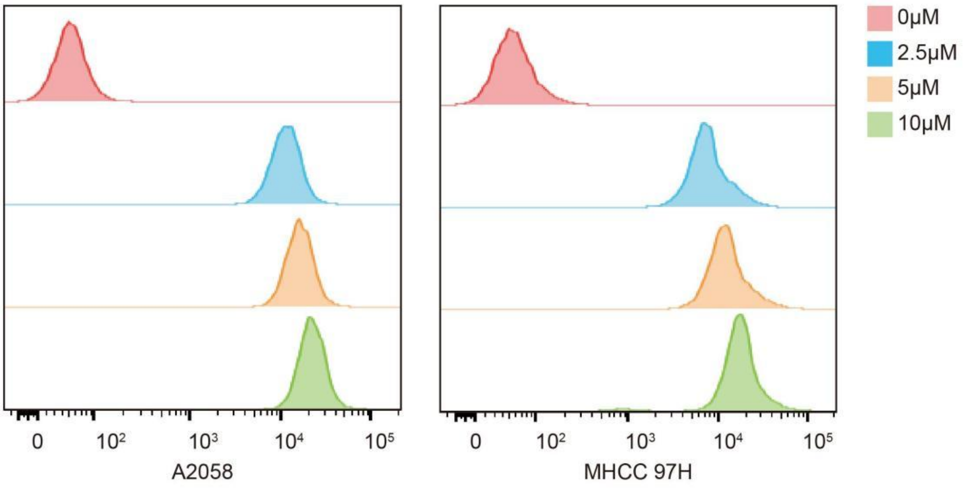

Figure S5. Quantitative analysis of T-T NPs in A2058 and MHCC 97H cells with concentration-dependence detected by FCM.

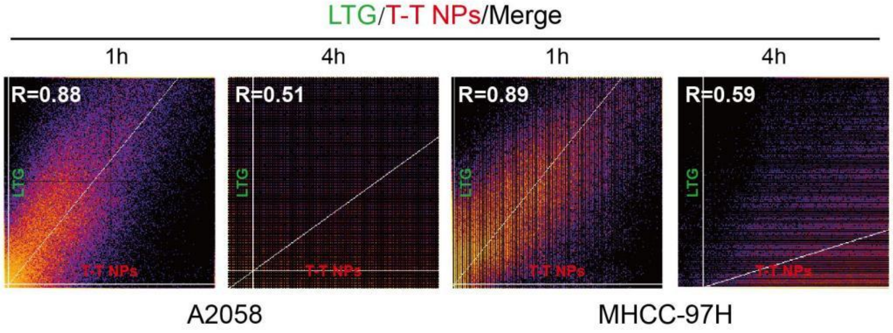

Figure S6. Co-localization quantitation analysis of A2058 cells and MHCC 97H cells stained with T-T NPs, LysoTacker Green (LTG) and MitoTracker Green (MTG).

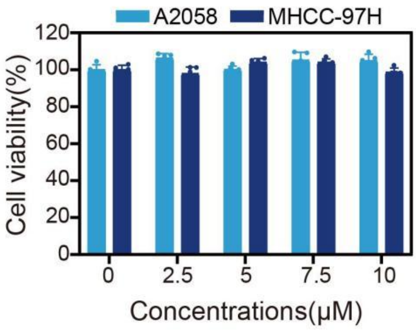

Figure S7. CCK-8 cell viability assay of A2058 and MHCC 97H cells treated with different concentration of T-T NPs without light irradiation under hypoxia.

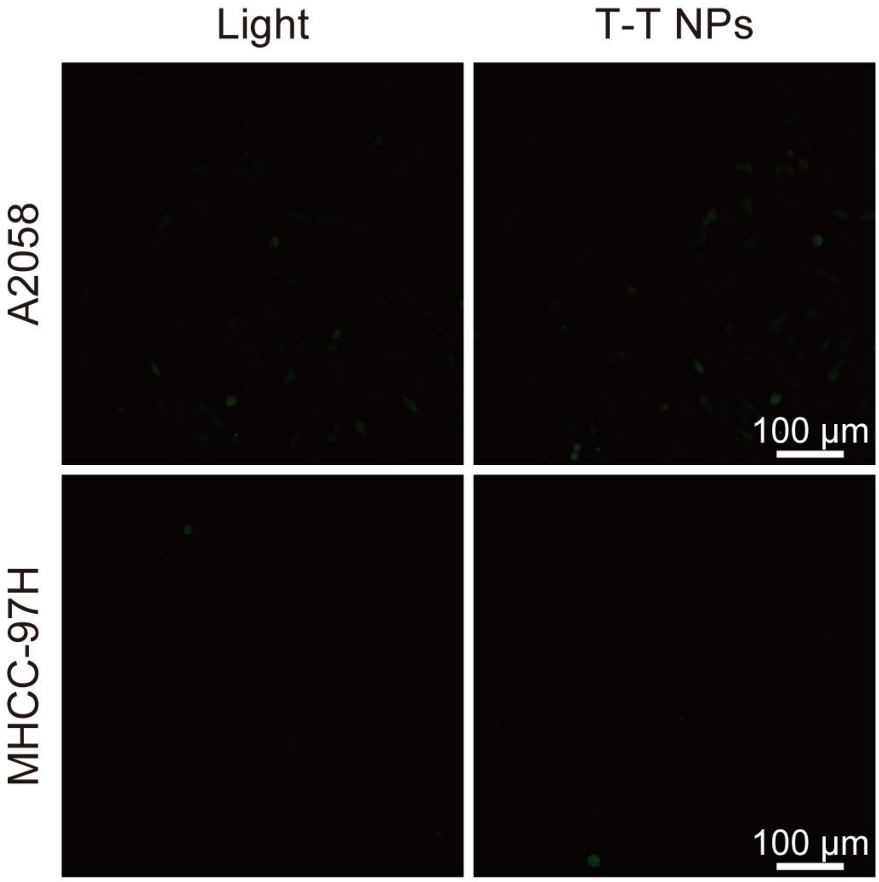

Figure S8. The intracellular ROS generation in light only group and T-T NPs only group using DCFH-DA (20 μM).

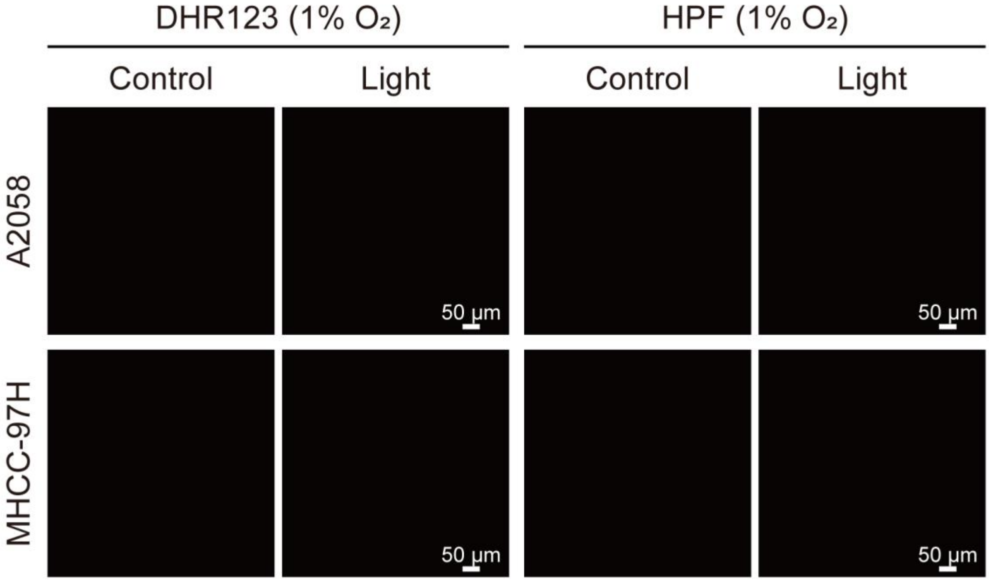

Figure S9. Intracellular $O_2^{\bullet -}$ and •OH detection using different commercial indicators DHR123 (20 μM) and HPF (20 μM) respectively, in control group and light only group under hypoxia.

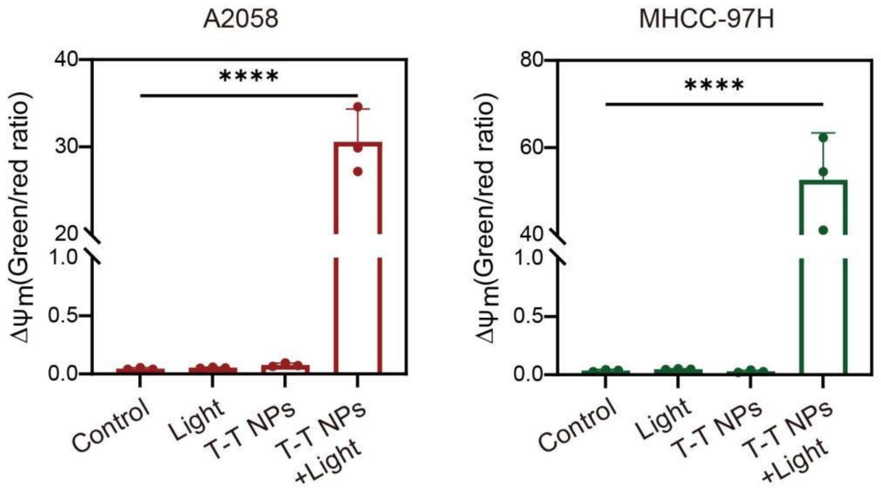

Figure S10. Mitochondrial membrane potential ($\Delta\psi_m$) data analysis of cells in different groups which were stained with JC-10. (****, $p < 0.0001$)

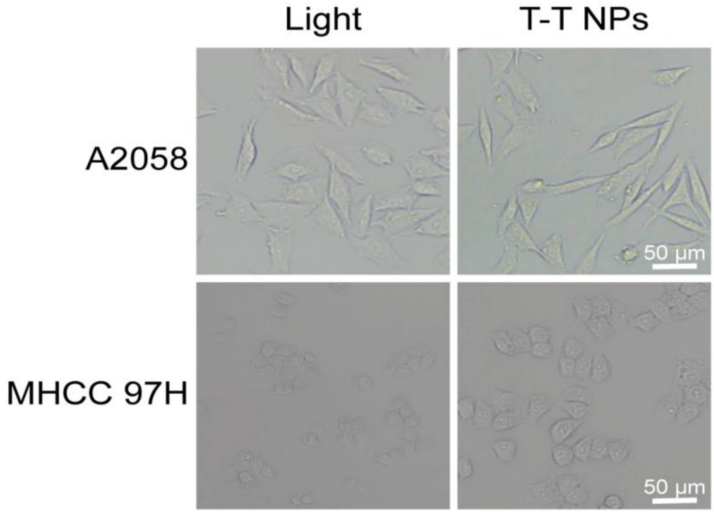

Figure S11. Representative images of A2058 and MHCC 97H cells morphology change after various treatments.

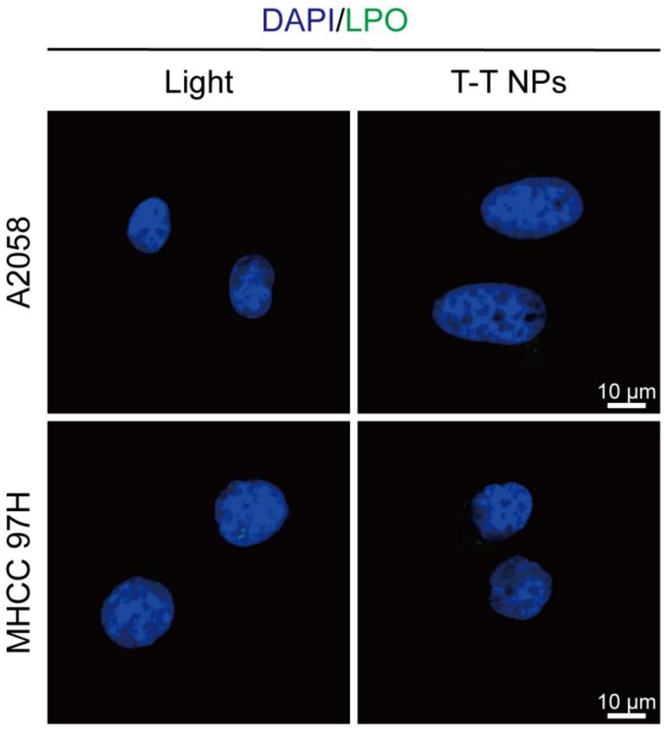

Figure S12. Confocal fluorescence images of lipid peroxidation using C11-BODIPY (581/591) dye.

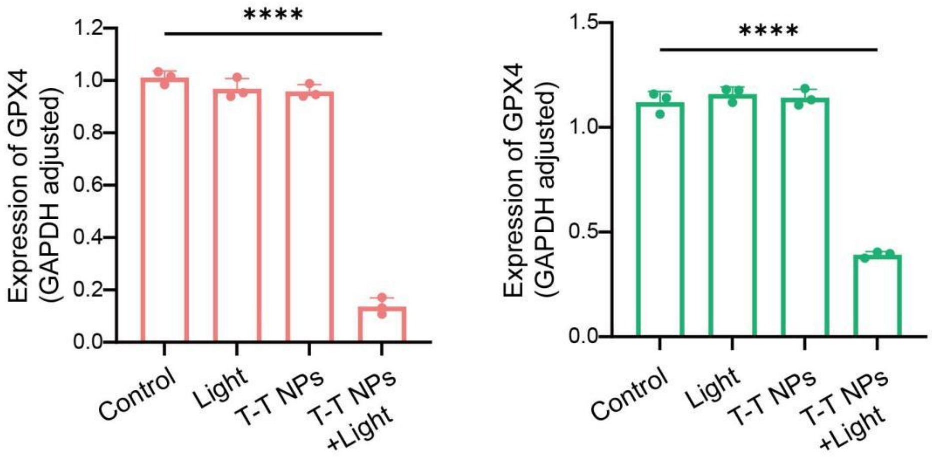

Figure S13. Semi-quantitative analysis of the protein levels of GPX4 in each group of A2058 and MHCC 97H cells. Bars are means ± standard error (n = 3). (****, $p < 0.0001$)

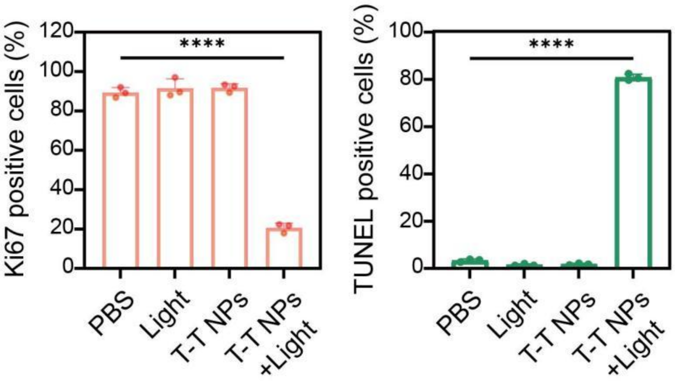

Figure S14. Quantitative analysis of Ki67 positive cell number and TUNEL positive cell number from the tumor tissues in different treatment groups. (****, $p < 0.0001$)

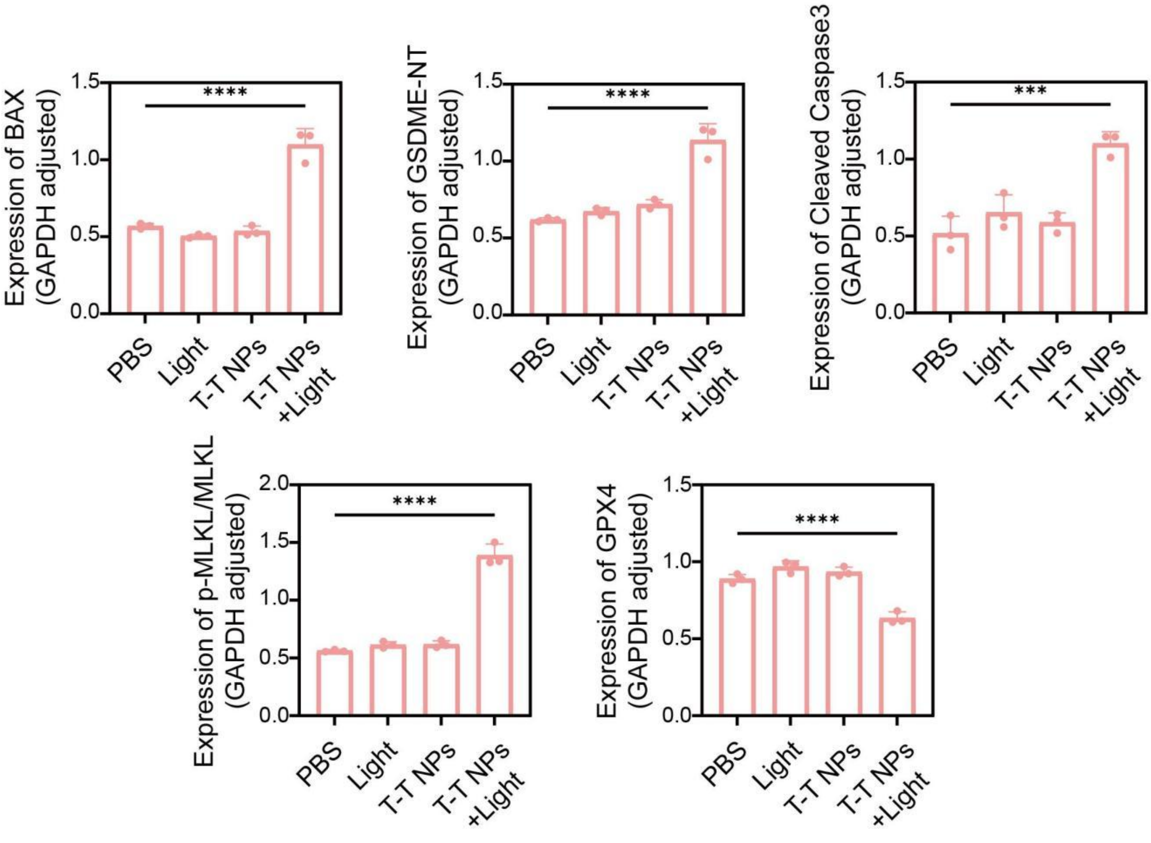

Figure S15. Semi-quantitative analysis of the protein levels of representative proteins in each group of tumor issues. Bars are means ± standard error (n = 3). (***, $p < 0.001$; ****, $p < 0.0001$)

References

1. Zhou T, Zhu J, Shang D, Chai C, Li Y, Sun H, et al. Mitochondria-anchoring and AIE-active photosensitizer for self-monitored cholangiocarcinoma therapy. Mater. Chem. Front., 2020,4, 3201-3208.
